# Influence of Sociodemographic, Premorbid, and Injury-Related Factors on Post-Concussion Symptoms after Traumatic Brain Injury

**DOI:** 10.3390/jcm9061931

**Published:** 2020-06-19

**Authors:** Marina Zeldovich, Yi-Jhen Wu, Anastasia Gorbunova, Ana Mikolic, Suzanne Polinder, Anne Marie Plass, Amra Covic, Thomas Asendorf, Nada Andelic, Daphne C. Voormolen, Nicole von Steinbüchel, on behalf of the CENTER-TBI 7 Participants and Investigators

**Affiliations:** 1 Institute of Medical Psychology and Medical Sociology, University Medical Center Göttingen, 37073 Göttingen, Germany; yi-jhen.wu@med.uni-goettingen.de (Y.-J.W.); anastasia.gorbunova@med.uni-goettingen.de (A.G.); annemarie.plass@med.uni-goettingen.de (A.M.P.); amra.covic@med.uni-goettingen.de (A.C.); nvsteinbuechel@med.uni-goettingen.de (N.v.S.); 2 Department of Public Health, Erasmus MC, University Medical Center Rotterdam, 3000 Rotterdam, The Netherlands; a.mikolic@erasmusmc.nl (A.M.); s.polinder@erasmusmc.nl (S.P.); d.voormolen@erasmusmc.nl (D.C.V.); 3 Department of Medical Statistics, University Medical Center Göttingen, 37073 Göttingen, Germany; thomas.asendorf@med.uni-goettingen.de; 4 Department of Physical Medicine and Rehabilitation, Oslo University Hospital, 0450 Oslo, Norway; nandelic@online.no; 5 Faculty of Medicine, Institute of Health and Society, Research Centre for Habilitation and Rehabilitation Models, 0373 Oslo, Norway

**Keywords:** RPQ, post-concussion symptoms, traumatic brain injury, negative binomial model, zero-inflated negative binomial model

## Abstract

Background: Post-concussion symptoms (PCS) are often reported as consequences of mild and moderate traumatic brain injury (TBI), but these symptoms are not well documented in severe TBI. There is a lack of agreement as to which factors and covariates affect the occurrence, frequency, and intensity of PCS among TBI severity groups. The present study therefore aims to examine the association between sociodemographic, premorbid, and injury-related factors and PCS. Methods: A total of 1391 individuals (65% male) from the CENTER-TBI study were included in the analyses. The occurrence, frequency (number of PCS), and intensity (severity) of PCS were assessed using the Rivermead Post-concussion Symptoms Questionnaire (RPQ) at six months after TBI. To examine the association between selected factors (age, sex, living situation, employment status, educational background, injury and TBI severity, and premorbid problems) and PCS, a zero-inflated negative binomial model (ZINB) for occurrence and frequency of PCS and a standard negative binomial regression (NB) for intensity were applied. Results: Of the total sample, 72% of individuals after TBI reported suffering from some form of PCS, with fatigue being the most frequent among all TBI severity groups, followed by forgetfulness, and poor concentration. Different factors contributed to the probability of occurrence, frequency, and intensity of PCS. While the occurrence of PCS seemed to be independent of the age and sex of the individuals, both the frequency and intensity of PCS are associated with them. Both injury and TBI severity influence the occurrence and frequency of PCS, but are associated less with its intensity (except “acute” symptoms such as nausea, vomiting, and headaches). Analyses focusing on the mTBI subgroup only yielded results comparable to those of the total sample. Discussion: In line with previous studies, the results support a multifactorial etiology of PCS and show the importance of differentiating between their occurrence, frequency, and intensity to better provide appropriate treatment for individual subgroups with different symptoms (e.g., multiple PCS or more intense PCS). Although PCS often occur in mild to moderate TBI, individuals after severe TBI also suffer from PCS or post-concussion-like symptoms that require appropriate treatment. The chosen statistical approaches (i.e., ZINB and NB models) permit an ameliorated differentiation between outcomes (occurrence, frequency, and intensity of PCS) and should be used more widely in TBI research.

## 1. Introduction

Traumatic brain injury. Traumatic brain injury (TBI) is defined as “an alteration in brain function, or other evidence of brain pathology, caused by an external force” (p. 1637) [1]. It can be caused by falls, assaults and domestic violence, accidents, sports, and other types of activities. TBI represents a considerable source of disability and death the world over, with a rather broad aggregated incidence rate of 369 (331–412) cases per 100,000 people per year and a population-based mortality rate ranging from 11.7 to 15.0 cases per 100,000 injured [2,3,4]. 

TBI severity can be classified into three groups using the Glasgow Coma Scale (GCS) [5]: mild (mTBI; GCS ≥ 13), moderate (9 ≤ GCS ≤ 12), and severe (GCS ≤ 8). Among all patients, 70 to 90 percent sustain a mild TBI (mTBI) [6,7], while 10 to 30 percent experience a moderate or severe TBI. Within the mTBI group, the distinction between complicated (with abnormalities in a computed tomography (CT) scan) and uncomplicated (without CT abnormalities) might be a useful criterion to further differentiate the impact of severity of mTBI [8]. 

Post-concussion symptoms. Post-concussion symptoms (PCS) commonly follow mild and moderate TBI [9], but some symptoms occur across all TBI severity groups, including severe TBI, e.g., cognitive deficits (forgetfulness) [10,11]. Complaints such as headaches, nausea, dizziness, sensitivity to light or noise, blurred or double vision, problems with concentration and memory, fatigue, insomnia, restlessness, irritability, and anxiety and depression typically occur within the first few days after TBI [12,13]. Furthermore, frequently reported PCS are headaches, fatigue, forgetfulness, and sleep disturbances [14,15]. Some studies report that PCS largely subside within two weeks and almost completely disappear after three months [14], while others report that they may remain for years after TBI [16,17]. Long-lasting PCS can impair the individuals’ daily life, their working ability, and self-reported health-related quality of life (HRQoL) [18]. Persistent PCS are often referred to as a post-concussion syndrome, diagnosed according to criteria of ICD-10 [13] or DSM-IV [12]. However, the concept of post-concussion syndrome has been questioned in the last decade [15,19,20,21], as indeed has the use of the term “concussion” [22]. Therefore, the present study focusses on reported symptoms without assuming a diagnostic entity. 

Factors associated with the development of PCS. Overall, researchers agree that the development of PCS after TBI has a multifactorial etiology that is associated with sociodemographic, premorbid, injury-related, post-morbid, and intrapersonal factors. Sex, age, educational background, and living situation seem to significantly influence the probability of developing PCS [15,16,23,24]. Research findings show that female sex and living alone are risk factors for the development of PCS, but there is no agreement on the association with age [25,26]. In order to adjust interventions, therapy, and health care, the question which sociodemographic factors contribute to a higher risk of experiencing PCS still needs further investigation.

Further, the presence of premorbid mental health problems (e.g., depression, anxiety, addiction, and others) [27,28,29] and previous concussions [30] are reported to have a significant association with the occurrence of PCS after TBI. Finally, neurological complications, loss of consciousness (LOC), post-traumatic amnesia (PTA), and hospitalization may have an impact on the occurrence and progression of PCS [16,20,24,26,31,32], though some studies have failed to find a significant impact of LOC [31] and PTA [33] on the occurrence of PCS. 

Most of the studies so far have applied a logistic regression to identify factors affecting the probability of PCS occurrence [18,29]. For this purpose, the total score of respective questionnaires is categorized into two groups: no occurrence of PCS and occurrence of PCS. This approach is statistically correct, but dichotomizing the data leads to a loss of information [34] and impedes the differentiation between occurrence and frequency (i.e., number) or intensity (i.e., severity) of PCS. Other research on PCS has used linear regression [11]. Linear models have strict assumptions and results could be biased due to skewed data distributions [35], which is often the case for the total scores of questionnaires. 

To shed some light on the controversies surrounding PCS after mTBI, Iverson [36] examined the multifactorial structure of PCS using a comprehensive network analysis. With this very promising approach, clusters of symptoms can be identified that relate to specific subgroups of individuals after TBI. However, the complexity of the method does not yet allow it to be applied easily in clinical routine.

Some studies show that PCS are not TBI-specific and occur in individuals with chronic pain [37], nonbrain injury patients [33], or even healthy populations [38]. In conclusion, there are still a lot of controversies about the etiology and development of PCS in both TBI and non-TBI populations. However, individuals from all TBI severity groups report suffering from PCS [11], so it is important to investigate this phenomenon further in order to provide appropriate treatment.

Study objectives. Given the lack of agreement among researchers concerning factors associated with the development of PCS after TBI, as well as the lack of differentiation between factors contributing to their occurrence, frequency, and intensity, the present study aims to examine sociodemographic, premorbid, and injury-related factors associated with PCS as reported in previous research in a large sample of individuals after TBI. Moreover, PCS have been investigated either using approaches leading to a loss of information or complex methods that cannot easily be implemented in both research and clinical practice. To overcome these limitations, the present study uses alternative approaches that have rarely been used in TBI research so far.

The present study has the following aims:

1. Investigating the probability of *occurrence* and the *frequency* (number) of PCS by applying a zero-inflated negative binomial model (see Statistical analyses).
2. Examining the factors associated with the *intensity* of PCS among individuals after TBI using a well-established statistical approach (i.e., negative binomial model; see Statistical analyses).

## 2. Methods

### 2.1. Participants 

Participants were included from the Collaborative European NeuroTrauma Effectiveness Research in TBI (CENTER-TBI) study, a prospective longitudinal nonrandomized observational study across the severity spectrum of TBI from 18 countries. All individuals participating in the CENTER-TBI study had access to the health care system independently of their employment status and were enrolled in the study following brain injury, provided they met the inclusion criteria. The inclusion criteria were a clinical diagnosis of TBI, clinical indication for CT scan, presentation within 24 h after injury to an emergency room (ER), admission ward (ADM), or intensive care unit (ICU), and written informed consent for study participation. A total of *N* = 4509 individuals after TBI participated in the CENTER-TBI core study between December 2014 and December 2017 [39,40].

For the present study, we focused on individuals over 16 years of age who had participated in the RPQ assessments six months after TBI. According to the study design, the time window for the outcome assessments was defined as six months (−1/+2 months) after TBI. The entire TBI severity spectrum (uncomplicated mTBI, complicated mTBI, moderate, and severe) was included in the study to gain a more comprehensive insight into PCS in these TBI groups. Thus, *N* = 1391 individuals were included in this study. Analyses focusing on individuals after uncomplicated mTBI and complicated mild TBI only are presented in the Online Supplementary Material. 

### 2.2. Ethical Approval

The CENTER-TBI study (EC grant 602150) was conducted in accordance with all relevant laws of the EU, where directly applicable or having a direct effect, and all relevant laws of the country in which the recruiting sites were located, including, but not limited to, the relevant privacy and data protection laws and regulations (the “Privacy Law”), the relevant laws and regulations on the use of human materials, and all relevant guidance relating to clinical studies from time to time in force including, but not limited to, the ICH Harmonised Tripartite Guideline for Good Clinical Practice (CPMP/ICH/135/95) (“ICH GCP”) and the World Medical Association Declaration of Helsinki entitled “Ethical Principles for Medical Research Involving Human Subjects”. The informed consent of the patients and/or their legal representative/next of kin was obtained accordingly to the local legislation for all patients recruited in the Core Dataset of CENTER-TBI and documented in the e-CRF.

### 2.3. Instruments

As most outcome instruments only existed in English, the questionnaires were translated into the respective 18 languages, and linguistically and psychometrically validated by von Steinbuechel and her team following a standard process [41]. 

Data were retrieved from the CENTER-TBI database using the data access tool NEUROBOT and core 2.0 final sample (May 2019).

Sociodemographic Data. These data contained information on sex, age in years, living situation, highest education level, and employment status at the time of enrollment in the study. 

Premorbid Health History. Medical histories were assessed at study enrollment, including information on preinjury medical illnesses and mental health problems. Depending on their health status prior to TBI, we classified individuals into seven groups: *none* (no premorbid problems), *physical* (mobility and physical limitations), *concussion* (previous concussion or TBI, also caused by sports and other activities), *migraines* (in individuals themselves or their families), *emotional/addiction* (anxiety, depression, sleep disorders, schizophrenia, substance abuse, or other mental problems), and *mixed* when individuals reported multiple premorbid problems. To examine a possible effect of different premorbid problems on the development of PCS, individuals suffering from psychological problems prior to TBI were used as a reference group.

Measures assessing injury-related factors. The severity of TBI was rated on the Glasgow Coma Scale (GCS) [5] and the presence of CT abnormalities on the first CT scans (*uncomplicated mild*, GCS ≥ 13 and no CT abnormalities; *complicated mild*, GCS ≥ 13 and CT abnormalities present; *moderate*, 9 ≤ GCS ≤ 12; and *severe* TBI, GCS ≤ 8). The GCS was determined within the first 24 h post-injury.

With the Injury Severity Score (ISS), trauma severity and polytrauma were evaluated by calculating the sum of the squares of the highest values of the three body regions measured by the Abbreviated Injury Scale score (AIS) [42]. The ISS ranges from 0 to 75, whereby higher scores indicate greater impairment. 

Loss of consciousness (LOC) was considered as a variable providing additional information on the post-injury state of the individuals. It was coded as follows: 0 (*no*), 1 (*yes/suspected*). The assessment occurred based either on self-report, testimony, clinical interview, or medical chart.

Rivermead Post-Concussion Symptoms Questionnaire (RPQ) [9] is a self-report questionnaire which comprises 16 items rated on a five-point Likert scale (from 0 = *not experienced at all* to 4 = *a severe problem*). The RPQ covers 16 possible symptoms after TBI: headaches, dizziness, nausea and/or vomiting, noise sensitivity, sleep disturbance, fatigue, irritability, depression, frustration, forgetfulness and poor memory, poor concentration, slow thinking, blurred vision, light sensitivity, double vision, and restlessness. Individuals are asked to evaluate each symptom over the last 24 h compared to the time before the injury 

The reference is the time before injury. Therefore, when calculating the sum score, the category *no more of a problem (than before)* is treated as 0. The total score ranges from 0 (no presence of symptoms) to 64 (most severe symptoms). The questionnaire is characterized by satisfactory psychometric properties [9], although its factorial structure has repeatedly been discussed [43,44]. 

With the RPQ total score, Eyres et al. [43] determined the intensity (i.e., severity) of PCS as an “acute” (RPQ-3) and a “post-acute” (RPQ-13) score. Eyres et al. investigated PCS three months after TBI and recommended, based on the results of Rasch analyses, splitting the RPQ total score into two scores: RPQ-3 (including *headaches*, *dizziness*, and *nausea and/or vomiting*) and RPQ-13 (including the rest of the items). Headaches, dizziness, and nausea and/or vomiting are typically reported immediately after injury [16]. In the present study, we used RPQ assessments six months post TBI. By then, the “acute” PCS should theoretically already have disappeared. However, all RPQ items are used in most studies investigating long-term consequences after TBI concerning PCS [27,44,45]. Therefore, to allow a better comparison with other studies and better differentiation, “acute” and “post-acute” PCS were analyzed separately in addition to the total score.

Additionally, we analyzed the frequency (i.e., number) of PCS reported by study participants. To assess this, a new variable was constructed which dichotomized item responses as 0 (*not experienced at all* and *no more of a problem than before*) and 1 (*a mild problem*, *a moderate problem*, and *a severe problem*). The cut-off value of 2 (i.e., mild problem or worse) corresponds to one of the options for assessment of symptom severity [25]. Finally, items were summed up to calculate the total number of reported PCS (*min* = 0 and *max* = 16).

### 2.4. Statistical Analyses 

The analyses followed a two-step strategy: (1) Estimation of the model to identify associations between occurrence and frequency of PCS and sociodemographic, premorbid, and injury-related factors; and (2) estimation of the model inspecting the association between symptom intensity and the factors mentioned above. 

The sociodemographic and injury-related characteristics of study participants were compared with those not included in the final study sample using a multidimensional chi-square test (nominal variables) or a Mann–Whitney *U*-test (total scores and counts).

### 2.5. Model for Occurrence and Frequency of PCS

**Analyses of Count Data**. Finite non-negative counts, such as the number of diseases or symptoms, hospital stays, or visits to a physician occur frequently in clinical outcome research. Count data often consist of a large number of zero observations (excess zeros) and overdispersion (the mean of the distribution is lower than its variance) and, because of this, a special statistical approach may be less biased than traditional regression [46]. The zero-inflated model [47] provides a valuable tool for dealing with count data containing excess zeros. The model estimation consists of two parts: the *zero* part estimates the probability of the nonoccurrence vs. occurrence of a behavior (i.e., absence vs. presence of PCS) and the *count* part assesses the mean frequency of a behavior (i.e., average number of PCS). The count part can be estimated using different underlying distributions. We used the negative binomial distribution, as it deals with overdispersion well [48]. Thus, a zero-inflated negative binomial model (ZINB) was implemented to identify associations between occurrence and frequency of PCS and selected factors. The main advantage of the ZINB is its ability to separately identify factors contributing to the probability of occurrence versus nonoccurrence of PCS and the development of multiple symptoms.

We use rootograms [49] to visualize the predictive goodness of the model using the number of observed and expected counts on a square-root scale presented by bars and a line, respectively. Those bars exceeding the zero line indicate an underestimation (i.e., the predicted values are lower than the observed values). When the bars fail to reach the zero line, the observed counts are higher than the predicted counts. The “warning limits” for deviations were suggested by Tukey [50] (a deviation of ±1 is permissible) and verified through bootstrapping [49].

### 2.6. Model for Intensity of PCS

Concerning the intensity of PCS, we calculated modified scores for the RPQ total score, RPQ-3, and RPQ-13 score by including the *no more of the problem* responses rated with 1 in the sum score to achieve greater congruence in model estimation. We applied a standard negative binomial model (NB) to examine associations between PCS intensity and sociodemographic, premorbid, and injury-related factors. The model selection procedure was the same as for the ZINB. 

**Model selection and model fit.** We only tested interactions between factors that had revealed contradictory results in previous research trying to identify differences between subgroups (i.e., age * sex, sex * LOC, sex * PTA). The stepwise AIC procedure was used to establish simplified models for all the analyses. A stepwise model selection was performed using Akaike’s information criterion (AIC) [51]. This is equivalent to using a liberal *p*-value (*p* < 0.157) for factors with 1 degree of freedom (df). 

The model fit for all estimated models was assessed using a likelihood ratio test. The log-likelihood (LogLik) value was used for statistical testing. A significant result (*p* < 0.05) indicates that the chosen model describes the data better than a null model without covariates. 

All analyses were performed with the R version 3.6.1 [52] and the packages *countreg* [53], *pscl* [54] and *MASS* [51] for the model estimations and rootograms. 

## 3. Results

### 3.1. Sample Characteristics

**Figure 1:**
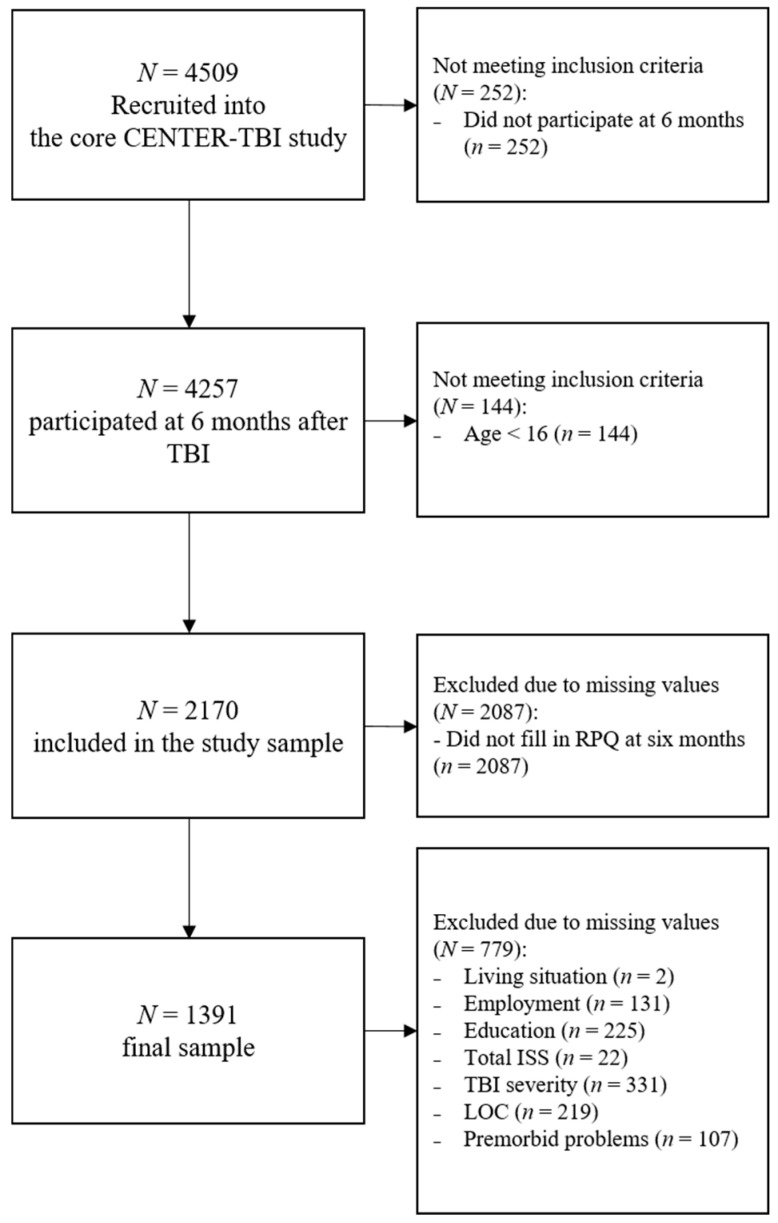
Attrition diagram of participants included in the study sample.

Our study sample consisted of *N* = 1391 individuals (65% male) of all TBI severities (for detailed sample attrition see Figure 1). 

Approximately half of individuals (49%) did not fill out the RPQ at six months after TBI. A comparison between individuals who had completed the RPQ and those who did not participate in the RPQ assessments showed significant differences in the TBI severity groups: 42% of those not included suffered from severe TBI, while the number of severely injured in the group included was 18%.

The amount of missing values across covariates varied from <1% (ISS) to 15% (TBI severity groups). The analyses of missing data revealed no systematic patterns. Therefore, we treated them as missing completely at random (MCAR) and used complete cases to ensure comparability between estimated models. 

Table 1 provides sample characteristics; the numbers in parenthesis are used for references within the text. Study participants (*n* = 1391) were compared with individuals who did not participate in the analyses (*n* = 779) with respect to each variable integrated in the models (including dependent variables). There were slightly more retired individuals among those not included (28% vs. 23%). They were mostly admitted to ICUs (43% vs. 39%), and had sustained a severe TBI more frequently (24%) compared to those included (17%). Additionally, those not included reported more intense “acute” RPQ symptoms.

**Figure 2:**
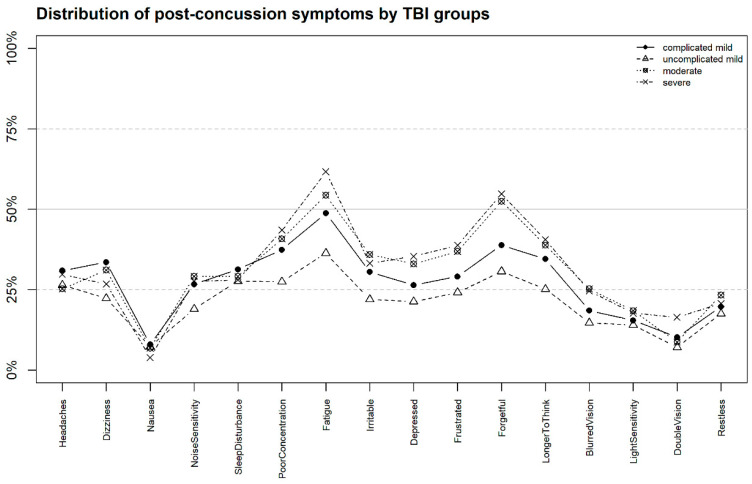
Amount of individual PCS rated as being at least mild (RPQ item score ≥ 2) by TBI severity group six months after traumatic brain injury (TBI).

In our study sample, 28% of the participants reported no PCS, whereas 58% sustained an uncomplicated mTBI, followed by complicated mTBI (29%), moderate (5%), and severe (8%). The proportion of individuals with PCS six months after TBI ranges from 7% (nausea) to 46% (fatigue). Half of the participants suffered from at least three symptoms (Q_25_ = 0, Q_75_ = 7). More than one third reported forgetfulness, poor concentration, and cognition problems (longer to think). About one fourth sustained headaches and dizziness six months after TBI. Figure 2 shows the proportion of individuals by TBI groups who rated PCS as being at least mild compared to the time before TBI. Again, fatigue achieved the highest amount (from 36% in the uncomplicated mTBI group to 62% in the severe group), followed by forgetfulness, especially in individuals after moderate and severe TBI (52% and 55%, respectively), and poor concentration (43% by severe, 40% by moderate, and 37% by complicated mTBI). The uncomplicated mTBI group showed the lowest level of PCS, except for nausea, compared with other groups.

### 3.2. Model Building Procedure

The initial model included the eleven independent variables listed in Table 1 (1–10). The dependent variables were the number of PCS (for occurrence and frequency of PCS) and various RPQ scores (for PCS intensity). All but two factors (living situation (3) and LOC (9)) were significant in at least one model. The difference between the model for the intensity of PCS and the model for the occurrence and frequency of PCS was that the patients’ strata (ER, ADM, and ICU) were included, whereas in the model for the occurrence and frequency of PCS, strata were insignificant. Additionally, the three RPQ scores differed in that TBI severity groups were only included in the model for the RPQ-3 score and showed no significance in both models for the RPQ total score and RPQ-13 score for chronic PCS. Furthermore, the ISS was not included in the model for the RPQ total score. Table 2 provides an overview on the factors associated with different types of outcome variables measuring PCS.

### 3.3. Occurrence and Frequency of PCS

**Figure A1:**
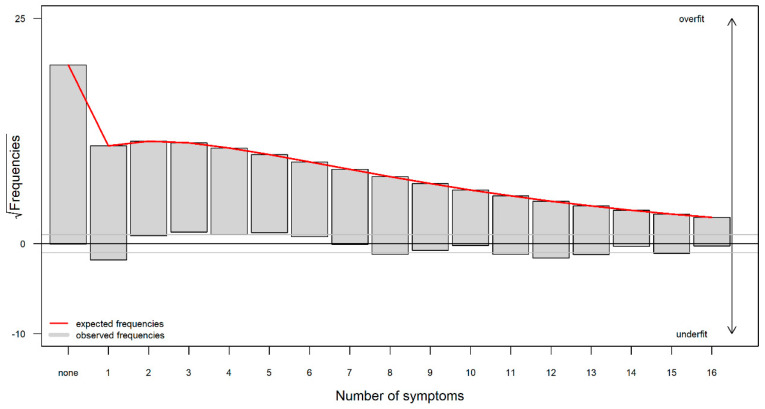
Goodness of fit (observed and expected counts on a square-root scale) of the ZINB model. The thin grey line (y = ±1) represents the reference indicating where the deviations between observed and expected counts are either critically over- or underestimated.

Goodness of fit. The rootogram in Figure A1 in Appendix A shows the goodness of fit of the estimated ZINB model. According to the rootogram, almost all deviations were in an acceptable range (±1); we observed only a slight underfit in counts 1 and 12. This indicates a good model fit. The model differed significantly from the null model without covariates (LogLik < 0.001, df = 43).

**Figure 3:**
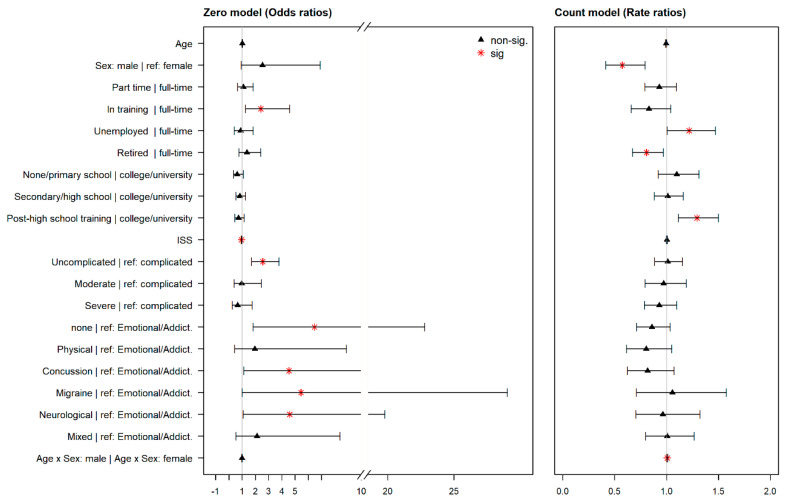
Odds ratios for the final ZINB model (the zero part) and rate ratios for the count part. Asterisks (*) identify factors, factor levels, and interactions that significantly influence the occurrence and the frequency of PCS. The zero model visualizes the probability of the nonoccurrence of the PCS, i.e., values < 1 mean that the probability of developing PCS is higher compared with the reference group (for nominal variables) or for higher values (for continuous variables). The count model visualizes the probability of developing more PCS on average, i.e., values < 1 mean that the probability of developing more PCS is lower compared with the reference group (for nominal variables) or for higher values (for continuous variables).

Contributing factors. According to model estimates, different factors contributed to the probability of occurrence of PCS and to their average number (i.e., frequency). Figure 3 provides a visualization of the model coefficients: odds ratios (OR) for the zero model (left part) and rate ratios (RR) for the count model (right part).

According to the zero model (probability of absence vs. presence of PCS), employment (4), ISS (7), TBI severity (8), and premorbid problems (10) contributed significantly to the probability of developing any PCS. The following factors and factor levels were associated with a higher risk of developing PCS: being full-time employed compared with being in training (4), having a higher ISS score (7), suffering from a complicated mTBI compared with an uncomplicated mTBI (8), and having suffered from psychological problems prior to TBI compared with reporting no problems, previous concussions (incl. TBI), migraines, or neurological problems. For more details, see Table A1 in Appendix B (left part, zero model).

The count model determined factors associated with a higher average number of PCS. Being female (2), unemployed (compared to full-time employees) (4), being full-time employed compared to being retired (4), being in post-high school training (compared to college or university graduates) (5) was associated with a higher number of PCS. Despite age (1) revealing no significance, the interaction term between age and sex was significant: with higher age, men tended to report more PCS on average than women. Figure 3 (right part) shows the rate ratios (RR) for the factors and factor levels considered.

Focusing on the uncomplicated mTBI and complicated mTBI subsamples, the same factors as in the total sample were found to contribute to the occurrence and frequency of PCS (see Table S1 in the Online Supplement).

### 3.4. Intensity of PCS

**Figure A2:**
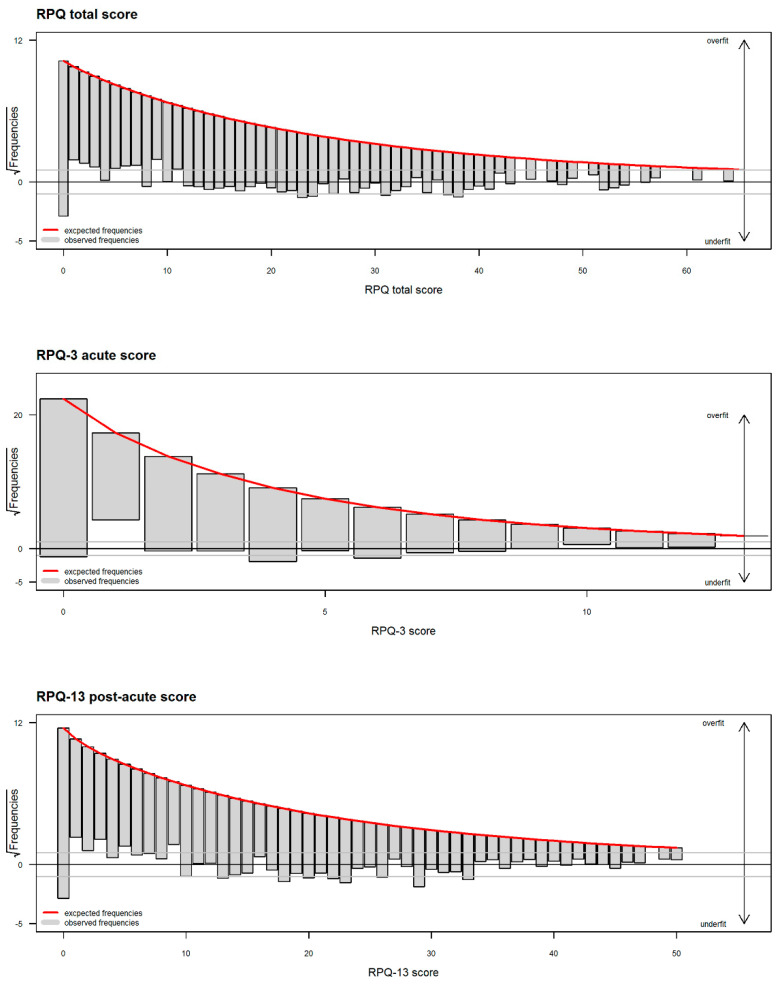
Goodness of fit (observed and expected counts on a square-root scale) of the negative binomial model (dependent variables: RPQ total score, RPQ-3, and RPQ-13 scores, respectively). The thin grey line (y = ±1) represents the reference indicating where the deviations between observed and expected counts are either critically over- or underestimated.

Goodness of Fit. According to the rootograms (Figure A2 in Appendix A), models considering RPQ total score and RPQ-13 score led to a slight underestimation of zero counts, which was expected since negative binomial models can deal well with overdispersion but reveal some problems in estimating excess zeros. Almost no deviations were outside of the “warning limits” (±1), indicating a good model fit. All three models performed better than a respective null model with *p* < 0.001.

**Figure 4:**
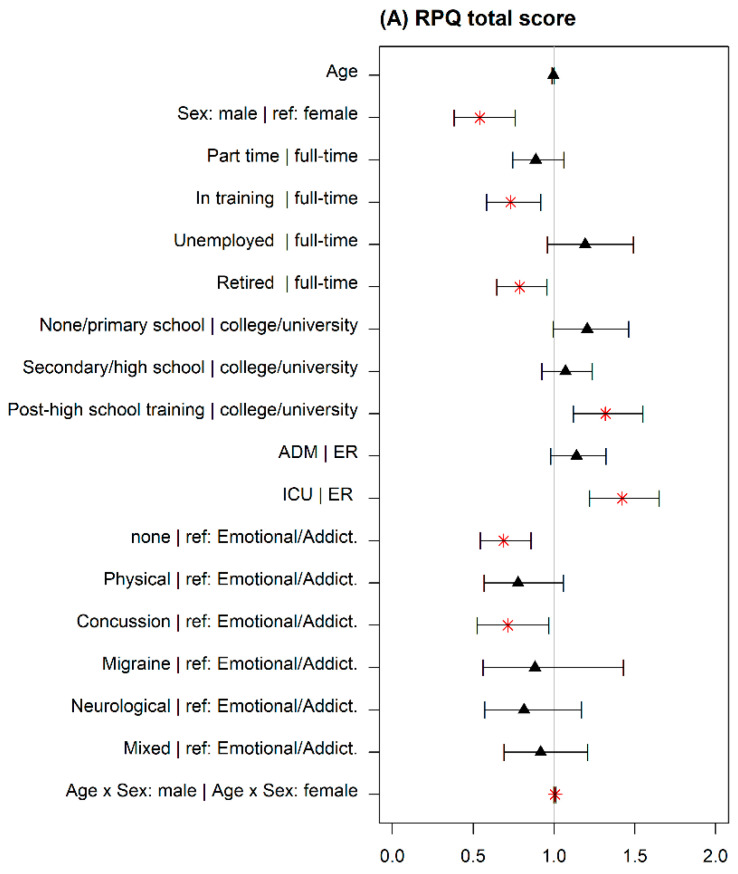

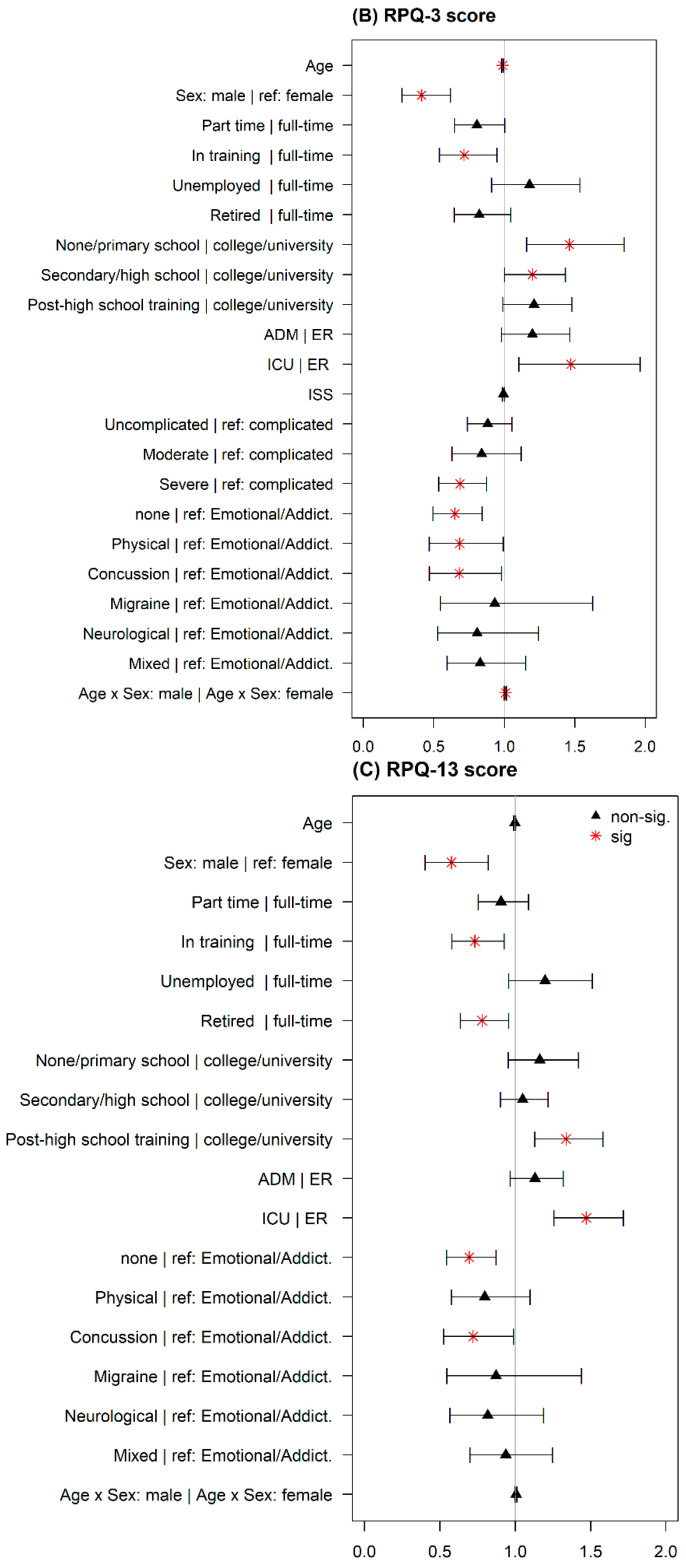
Rate ratios for the final negative binomial (NB) model for the (**A**) RPQ total score, (**B**) RPQ-3 acute score, and (**C**) the RPQ-13 post-acute score. Asterisks (*) identify factors, factor levels, and interactions that significantly influence the occurrence and the frequency of PCS. Values < 1 mean that the probability of developing more PCS is lower compared with the reference group (for nominal variables) or for higher values (for continuous variables).

Contributing factors. Factors associated with the intensity of PCS differed for the total score (RPQ), the acute (RPQ-3), and post-acute scores (RPQ-13) as shown in Table 2. Higher intensity of PCS was associated with the following factors: lower age (1) (RPQ-3), female sex (2), being full-time employed compared with being part-time employed (RPQ-3), in training, or retired (all but RPQ-3) (4), being in post-high school training (all but RPQ-3), or completing a primary school or a secondary school degree (RPQ-3) compared with college or university graduates (5). Furthermore, being admitted to an ICU compared to ER (6), having experienced a complicated mTBI compared to severe TBI (8), and having premorbid psychological problems prior to TBI (10) led to a higher probability of developing more intense PCS. Additionally, we observed an interaction between age and sex (RPQ total and RPQ-3): with increasing age, the intensity of PCS was higher in men compared with women (see Figure 4A–C; for model coefficients, see Table A2 in Appendix B).

The results for the uncomplicated mTBI and complicated mTBI subsamples were comparable with the total sample. With one exception, individuals without any intercranial abnormalities revealed a lower probability of developing more intense “acute” PCS compared to those with abnormal CT findings. There were no associations between PCS intensity and the hospital location where patients were admitted: ER, ward, or ICU. Moreover, LOC, as well the interaction term between LOC and sex, were included in the models for the RPQ total score and RPQ-13 score according to the AIC stepwise procedure, but without a significant effect (*p* > 0.05). For more details, see Table S2 in the Online Supplementary Material. 

## 4. Discussion

The present study aimed to examine the occurrence of PCS, their frequency, and intensity in association with sociodemographic, premorbid, and injury-related factors using an alternative statistical approach that minimizes loss of information. We therefore focused on factors frequently associated with PCS (age, sex, living situation, sociodemographic status, TBI severity, and premorbid physical, neurological, and psychological problems) [15,16,25,26,27,28,29,30]. Additionally, we used information on the type of admission to the hospital (ER, ADM, and ICU), and the total injury severity score (ISS). 

We performed statistical approaches suitable for handling data with excess zeros and overdispersion to examine the occurrence and frequency of PCS, and negative binomial distributions to identify associations between factors and the intensity of PCS. The ZINB model allows a differentiation between occurrence and frequency of outcomes. A combination of ZINB and NB allows a more nuanced insight into the development of PCS, their frequency, and intensity compared to previous approaches. 

The analyses were based on all TBI severity groups to provide a better differentiation while controlling for sociodemographic, injury-related, and premorbid factors. Although PCS commonly occur and are most commonly assessed in mild to moderate TBI groups, individuals after severe TBI also report PCS, in line with previous research [10,11]. Our results from the analyses of the mTBI subgroups differed only slightly from those of the total sample and the severe TBI group, showing that the same factors contributed to the occurrence, frequency, and intensity of PCS across all TBI severity groups.

Overall, while the results of the present study correspond to previous research findings, they also provide novel insights. Sociodemographic, premorbid, injury-related, and postmorbid factors are significantly associated with the development of PCS, supporting the concept of a multifactorial etiology as reported by Iverson [36]. 

Among the novel findings of this study is the importance of distinguishing between the investigated outcome types, as different factors influence the probability of occurrence of PCS, their frequency, and intensity. Moreover, different factors contribute to “acute” and “post-acute” PCS, as defined by Eyres and her colleagues [43]. 

The probability of the occurrence of PCS as well as factors associated with PCS have so far been the central focus of research [11,29,36]. Our findings allow the simultaneous consideration of the occurrence of PCS and their frequency, due to application of the ZINB model. On one hand, our results show that the probability of occurrence of PCS is associated with preinjury and injury-related factors. Premorbid psychological problems influence the development of PCS. In addition, both, TBI severity and overall extracranial trauma severity (polytrauma) are associated with a higher probability of developing PCS. The latter could explain why PCS also occur in general trauma populations, e.g., more severe extracranial injuries may lead to different symptoms and functional impairments and the development of post-concussion-like conditions. Further, individuals after complicated mTBI are more likely to develop PCS compared with individuals sustaining an uncomplicated mTBI. These results point to the need for better differentiation between the uncomplicated and complicated mTBI groups in research and clinical practice. First steps have been already taken within the present study (see Online Supplement). Recent studies contribute to an increasing knowledge on this topic.

On the other hand, in line with previous research based predominantly on logistic regression analyses [18,35,36], we have shown here that both frequency and intensity of PCS are associated with multiple factors such as sociodemographic (age, sex, employment, and education), premorbid (psychological problems prior to TBI), and injury-related factors (injury and TBI severity, admission type). This might be of great interest for clinicians as well as for those affected, as the choice, duration, and intensity of further treatment (medical, psychiatric, psychological, and physiotherapeutic) depend thereon. 

Additionally, complicated mTBI leads to more intense “acute” PCS than severe TBI, but not moderate or uncomplicated mTBI. This might be explained by the fact that individuals after severe TBI may be less aware of their deficits, may under-report symptoms during the acute phase [10], or their focus of attention is on other symptoms. Nonetheless, individuals after severe TBI reported a relatively high number of PCS, although it is not clear whether this was directly related to TBI or in combination with other extracranial injuries. To better differentiate between PCS caused by TBI and post-concussion-like symptoms caused by other factors (e.g., face-/neck or other physical trauma, emotional distress, and other life stressors related to injury), it will be necessary to distinguish between individuals after severe TBI with and without polytrauma.

On one hand, previous studies have shown that higher age is associated with a higher PCS risk [26]. Yet, Voormolen et al. (2018) have reported [25] no association between age and PCS development. Our results demonstrate a negative association between age and “acute” PCS. One of the possible explanations is that the study participants were 16 years of age or older. Pediatric studies on TBI and repetitive TBI have shown younger patients (<24 years of age) to be more vulnerable to TBI and its sequelae compared to adults because of the process of white matter myelination as a part of brain maturation [55]. Therefore, more PCS may be reported in combination with longer-lasting neurocognitive impairments [56,57]. Overall, 195 (14%) individuals participating in our study were under 24 years of age. They tended to report more (one symptom more on average) and more intense (on average two points more measured by RPQ total score) PCS compared to older individuals, which is in line with previous studies [55,56,57]. Thus, the negative association between age and “acute” PCS could be explained by this. We therefore recommend a more specific age differentiation when investigating PCS.

Moreover, we found age and sex to have a significant impact on the frequency of PCS. Our results suggest that the interaction term between age and sex should be considered when predicting the “acute” PCS (headaches, dizziness, and nausea/vomiting). This finding could probably explain contradictory results concerning the influence of age in previous research. With increasing age, men tended to report more PCS compared with women. This may also be related to changes in the endocrinal levels in both males and females. With increasing age, these changes may influence mood, strength, and quality of life [58,59] and recovery processes after TBI [60], and thus may lead to the observed differences between the sexes. Changes in social roles with aging may have an impact on the experiencing of PCS [61]. Previous research has not reported any information on the interaction between age and sex. A systematic review [62] including 77 papers on age and sex in persisting PCS concludes that older age and female sex have a significant impact on the development of the PCS. However, none of the studies provided information on the interaction between age and sex. Thus, conclusive evidence is needed here. Therefore, we support the idea that clinicians should consider this aspect more carefully when treating older individuals after TBI, and develop customized treatments and rehabilitation programs. Further research is required on sex and age differences and comparisons between different age groups. 

Additionally, premorbid psychological problems (addiction problems and emotional and mental health disorders existing prior to TBI) show a significant impact on the frequency of PCS after TBI, which is in line with previous research [27,28,29]. This finding indicates that a premorbid vulnerable population will benefit especially from appropriate psychiatric and psychological diagnostics, tailored treatments, and regular follow-ups.

In line with previous reviews [15], lower educational level (primary or secondary school degree) is associated with a higher intensity of acute PCS. This finding is also confirmed by healthy populations: people with higher education may have a better understanding of what health care services are available and how to use them. For this reason, they may perceive and report fewer symptoms [63].

In correspondence with previous research [64], fatigue is the most frequently reported PCS, with 46% incidence even six months after a TBI, often accompanied by headaches [65], sleep problems [66], and depression [67]. Fatigue itself can partly explain the presence of headaches, which are declared to be acute PCS but remain a problem for a relatively large group of participants (29%) six months post TBI. Fatigue is also the most frequently reported symptom in our severe TBIs group (62%), and the least frequent in those with uncomplicated TBI (36%) suggesting that TBI-related neuroanatomical brain injuries might contribute to the development of fatigue post-injury (i.e., neurogenic fatigue [68]). Further, cognitive symptoms (poor concentration, forgetfulness, longer time to think) are more pronounced in those with severe (and moderate) TBI, probably due to the condition-specific brain injuries. Nonetheless, more research is needed before the use of the RPQ can be recommended as a short screener of PCS also after severe TBI.

Previous studies have reported that premorbid variables such as emotional problems, pre-existing fatigue, and certain personality traits may also contribute to the vulnerability for the development of fatigue following TBI [69]. This emphasizes the need for appropriate differential clinical diagnostics, patient education and targeted long-term treatments, regardless of the factors that contribute to the condition. Yet here too, more knowledge is required concerning the mechanisms of fatigue after TBI, and short- and long-term fatigue trajectories, as well as effective treatment strategies.

Interestingly, and in contrast to previous studies on both TBI and non-TBI populations [15,25], living situation appears to have no significant impact either on the occurrence, or on the frequency or intensity of PCS. 

Study value. The present study has several strengths. First, the results are based on a large multicenter sample from 18 European countries across the severity spectrum of TBI. Second, it examines the probability of the occurrence, frequency, and intensity of PCS, which have not been investigated in a combined fashion so far. Statistical approaches applied in the study (the ZINB model in combination with NB model) have not yet been widely used in TBI research in particular. The ZINB has proven a preferable method when investigating the etiology of occurrence and frequency of PCS, whereas the application of NB is recommended when dealing with overdispersed data such as total scores of questionnaires. For clinical implications, please see below. 

Limitations of study. The validity of the RPQ, especially its factorial structure, has often been questioned [43,70]. Some studies show that the RPQ does not comprise one dimension. Therefore, we investigated the “acute” and the “post-acute” PCS separately, with a satisfactory discriminative added value. Furthermore, it has been criticized that the RPQ fails to cover all possible PCS and therefore needs to be extended [45]. Having been developed for assessing PCS in mild and moderate TBIs [9], its sensitivity and specificity in severe TBI may be limited. Yet, in this study, the RPQ captured symptoms that might be specific to severe TBI (such as fatigue, cognitive symptoms, double vision). Given the lack of research in the field of PCS in individuals after severe TBI, further research is needed.

Comparing individuals involved in the analyses with those who were not reveals differences in several variables that are integrated into the models. This can be partly explained by TBI severity: the individuals omitted sustained a severe TBI significantly more often and were more likely to have been admitted to ICUs. 

## 5. Conclusions

Although PCS often occur in mild to moderate TBI, individuals after severe TBI also develop PCS requiring appropriate assessment, diagnostics, and treatment. To provide further evidence on whether PCS are TBI-specific or not, more detailed comparisons between TBI and non-TBI populations are needed.

For clinical practice, the present study indicates the need for medical, psychiatric, and psychological diagnostics, and short- and long-term treatment, care, and rehabilitation, especially for individuals with psychological premorbid problems, men and women in different age groups, and individuals after intracranial injuries to ensure a successful return to everyday life after TBI.

## Figures and Tables

**Table 1:** Descriptive statistics of the study sample.

No.	Variable	Groups (*Reference Group in Italic*)	*N*	%	*M (SD)*
(1)	Age ^a^	-	1391	100%	48.75 (19.22)
(2)	Sex	*female*	483	35%	-
		male	908	65%	-
(3)	Living situation	*alone*	292	21%	-
		not alone	1099	79%	-
(4)	Employment	*full-time*	634	46%	-
		part time	184	13%	-
		in training	145	10%	-
		unemployed	107	8%	-
		retired	321	23%	-
(5)	Education	none/primary school	197	14%	-
		at least secondary/high school	506	36%	-
		post-high school training	298	21%	-
		*college/university*	390	28%	-
(6)	Stratum	*ER*	320	23%	-
		Admission	520	37%	-
		ICU	551	40%	-
(7)	ISS ^a^	-	1391	100%	18.13 (14.62)
(8)	TBI severity	uncomplicated mild	564	41%	-
		*complicated mild*	492	35%	-
		moderate	103	7%	-
		severe	232	17%	-
(9)	LOC	no	456	33%	-
		*yes*	935	67%	-
(10)	Premorbid problems	none	903	65%	-
		physical	89	6%	-
		concussion	94	7%	-
		migraine	25	2%	-
		neurological	53	4%	-
		*emotional or addiction*	90	6%	-
		mixed	137	10%	-
(-)	No. PCS ^a^	-	1391	100%	4.15 (4.28)
	RPQ Total score ^a^	-	1391	100%	13.57 (12.37)
	RPQ-13 score ^a^	-	1391	100%	11.54 (10.71)
	RPQ-3 score ^a^	-	1391	100%	2.02 (2.41)
	Total		1391	100%	

*Note*. Numbers in parenthesis are used for references within the text and further tables; ^a^ for continuous variables and the total scores, mean (M) and standard deviation (SD) are reported; ISS = total injury severity score, TBI = traumatic brain injury, LOC = loss of consciousness, PCS are evaluated with the frequency (symptom rated at least as mild), modified RPQ total score, RPQ-13, and RPQ-3 scores (considering “1” responses).

**Table 2:** Overview of factors associated with occurrence, frequency, and intensity of PCS at six months after TBI.

		Post-Concussion Symptoms				
No.	Variable	Occurrence ^a^	Frequency ^b^	Intensity ^c^	Intensity Acute ^d^	Intensity Post-Acute ^d^
(1)	Age	+−	+−	+−	++	+−
(2)	Sex	+−	++	++	++	++
(1:2)	Age: Sex	+−	++	++	++	+−
(3)	Living situation	−−	−−	−−	−−	−−
(4)	Employment	++	++	++	++	++
(5)	Education	+−	+−	++	++	++
(6)	Stratum (ER, ADM, ICU)	−−	−−	++	++	++
(7)	Total ISS	++	+−	−−	+−	−−
(8)	TBI severity	++	+−	−−	++	−−
(9)	LOC	−−	−−	−−	−−	−−
(10)	Premorbid problems	++	+−	++	++	++

*Note*. ++ = significant association (at least one factor level), + = weak association but included in the model, −− = no association and not included in the model; Age: Sex = interaction term; ^a^ Occurrence of PCS is measured by number of RPQ symptoms in the zero part of the ZINB model; ^b^ Frequency of PCS is measured by number of RPQ symptoms in the count part of the ZINB model; ^c^ Intensity of the post-concussion symptoms is measured by the modified RPQ total score, i.e., 1 is treated as 1 and not as 0 as proposed by King et al. (1995); ^d^ Intensity of the acute and post-acute PCS is measured by the modified RPQ-3 and RPQ-13 total scores, respectively, i.e., 1 is treated as 1 and not as 0 as proposed by Eyres et al. (2007).

## Supplementary Materials

The following materials are available online at https://www.mdpi.com/2077-0383/9/6/1931/s1, Table S1: Estimates, odds ratios and rate ratios of the ZINB model. The zero model predicts the probability of non-occurrence of PCS; the count model predicts the average number of PCS for the mTBI subsample. Table S2: Estimates and rate ratios of negative binomial model for intensity of PCS evaluated with the RPQ total score, RPQ-3, and RPQ-13 score for the mTBI subsample. List of the CENTER-TBI participants and investigators.

## Appendix B

**Table A1:** Estimates, odds ratios, and rate ratios of the ZINB model. The zero model predicts the probability of nonoccurrence of PCS; the count model predicts the average number of PCS.

			Zero Model						Count Model					
No.	Predictors	Levels|*reference group*	Est.	S.E.	*z*	OR	CI_95_	*p*	Est.	S.E.	*z*	RR	CI_95_	*p*
		Intercept	−3.62	0.88	−4.12	0.03	[0.01, 0.15]	**<0.001**	2.06	0.20	10.21	7.88	[5.30, 11.71]	**<0.001**
(1)	Age	in years	0.01	0.01	1.41	1.01	[0.99, 1.03]	0.158	0.00	0.00	−1.24	1.00	[0.99, 1.00]	0.216
(2)	Sex	male|*female*	0.93	0.51	1.81	2.53	[0.93, 6.90]	0.070	−0.56	0.17	−3.35	0.57	[0.41, 0.79]	**0.001**
(4)	Employment	part time|*full-time*	0.09	0.26	0.36	1.10	[0.66, 1.84]	0.722	−0.07	0.08	−0.86	0.93	[0.79, 1.10]	0.390
		in training|*full-time*	0.88	0.33	2.66	2.41	[1.26, 4.61]	**0.008**	−0.19	0.12	−1.61	0.83	[0.66, 1.04]	0.107
		unemployed|*full-time*	−0.12	0.37	−0.33	0.89	[0.43, 1.83]	0.743	0.20	0.10	2.02	1.22	[1.01, 1.47]	**0.044**
		retired|*full-time*	0.31	0.29	1.08	1.37	[0.77, 2.42]	0.280	−0.21	0.09	−2.30	0.81	[0.67, 0.97]	**0.022**
(5)	Education	none/primary school|*college/university*	−0.46	0.28	−1.62	0.63	[0.36, 1.10]	0.105	0.09	0.09	1.04	1.10	[0.92, 1.31]	0.298
		secondary/high school|*college/university*	−0.19	0.21	−0.89	0.83	[0.54, 1.26]	0.372	0.01	0.07	0.18	1.01	[0.88, 1.16]	0.861
		post−high school training|*college/university*	−0.31	0.23	−1.33	0.73	[0.47, 1.16]	0.182	0.26	0.08	3.37	1.29	[1.11, 1.50]	**0.001**
(7)	Total ISS	total injury severity score	−0.04	0.01	−3.22	0.96	[0.94, 0.98]	**0.001**	0.00	0.00	1.31	1.00	[1.00, 1.01]	0.190
(8)	TBI	uncomplicated mild|*complicated mild*	0.94	0.20	4.64	2.56	[1.72, 3.80]	**<0.001**	0.01	0.07	0.15	1.01	[0.89, 1.15]	0.879
		moderate|*complicated mild*	−0.02	0.47	−0.05	0.98	[0.39, 2.45]	0.964	−0.03	0.10	−0.28	0.97	[0.79, 1.19]	0.779
		severe|*complicated mild*	−0.40	0.49	−0.81	0.67	[0.26, 1.75]	0.415	−0.07	0.08	−0.86	0.93	[0.79, 1.10]	0.391
(10)	Premorbidproblems	none|*emotional*	1.87	0.64	2.90	6.47	[1.83, 22.81]	**0.004**	−0.15	0.10	−1.59	0.86	[0.71, 1.04]	0.112
		physical|*emotional*	0.68	0.77	0.88	1.97	[0.44, 8.88]	0.378	−0.22	0.14	−1.60	0.80	[0.61, 1.05]	0.110
		concussion|*emotional*	1.51	0.71	2.14	4.54	[1.13, 18.19]	**0.033**	−0.20	0.14	−1.46	0.82	[0.62, 1.07]	0.145
		migraine|*emotional*	1.69	0.85	1.98	5.44	[1.02, 29.05]	**0.048**	0.06	0.20	0.27	1.06	[0.71, 1.58]	0.785
		neurological|*emotional*	1.53	0.74	2.06	4.61	[1.07, 19.78]	**0.040**	−0.04	0.16	−0.22	0.97	[0.70, 1.32]	0.825
		mixed|*emotional*	0.75	0.70	1.07	2.12	[0.54, 8.39]	0.283	0.01	0.12	0.05	1.01	[0.80, 1.27]	0.962
(1:2)	Age:sex	age:male|*age: female*	−0.01	0.01	−1.08	0.99	[0.97, 1.01]	0.282	0.01	0.00	2.09	1.01	[1.00, 1.01]	**0.036**

*Note*. Numbers in parenthesis correspond to the numbers in Table 1 and are used for reference in the text; ER = emergency department, ADM = admission, ICU = intensive care unit, TBI = traumatic brain injury, age: sex = interaction between the age and sex groups. The zero model predicts the nonoccurrence of PCS; the count model predicts the average number of PCS. Est. = estimate, model coefficient, S.E. = standard error, *z* = z-value, OR = Odds ratio, CI_95_ = 95% confidence interval [lower, upper]; RR = rate ratio; **bold** *p*-values are significant at α = 0.05.

**Table A2:** Estimates and rate ratios of negative binomial model for intensity of PCS evaluated with the RPQ total score, RPQ-3, and RPQ-13 score.

			RPQ Total Score						RPQ-3 Score						RPQ-13 Score					
No.	Predictors	Levels|*Reference Group*	Est.	S.E.	*z*	*RR*	CI_95_	*p*	Est.	S.E.	*z*	*RR*	CI_95_	*p*	Est.	S.E.	*z*	*RR*	CI_95_	*p*
		Intercept	3.07	0.20	15.18	21.60	[14.36, 32.76]	**<0.001**	1.80	0.26	7.02	6.07	[3.63, 10.21]	**<0.001**	2.82	0.21	13.42	16.70	[10.94, 25.74]	**<0.001**
(1)	Age	in years	0.00	0.00	−1.33	1.00	[0.99, 1.00]	0.184	−0.01	0.00	−2.82	0.99	[0.98, 1.00]	**0.005**	0.00	0.00	−0.90	1.00	[0.99, 1.00]	0.366
(2)	Sex	male|*female*	−0.61	0.17	−3.59	0.54	[0.38, 0.76]	**<0.001**	−0.88	0.20	−4.32	0.41	[0.27, 0.62]	**<0.001**	−0.55	0.18	−3.09	0.58	[0.40, 0.82]	**0.002**
(4)	Employment	part time|*full-time*	−0.12	0.09	−1.32	0.89	[0.75, 1.06]	0.186	−0.22	0.11	−1.95	0.81	[0.65, 1.00]	0.052	−0.10	0.09	−1.07	0.90	[0.75, 1.09]	0.285
		in training|*full-time*	−0.31	0.12	−2.72	0.73	[0.58, 0.92]	**0.007**	−0.33	0.14	−2.35	0.72	[0.54, 0.95]	**0.019**	−0.31	0.12	−2.61	0.73	[0.58, 0.93]	**0.009**
		unemployed|*full-time*	0.18	0.11	1.56	1.19	[0.96, 1.49]	0.119	0.16	0.13	1.23	1.18	[0.91, 1.54]	0.218	0.18	0.12	1.55	1.20	[0.96, 1.51]	0.122
		retired|*full-time*	−0.24	0.10	−2.39	0.79	[0.65, 0.96]	**0.017**	−0.20	0.12	−1.60	0.82	[0.65, 1.05]	0.111	−0.25	0.10	−2.39	0.78	[0.64, 0.96]	**0.017**
(5)	Education	none/primary school|*college/university*	0.19	0.10	1.93	1.21	[1.00, 1.46]	0.054	0.38	0.12	3.23	1.46	[1.16, 1.85]	**0.001**	0.15	0.10	1.50	1.16	[0.95, 1.42]	0.135
		at least secondary/high school|*college/university*	0.07	0.07	0.92	1.07	[0.93, 1.24]	0.360	0.18	0.09	1.99	1.20	[1.00, 1.43]	**0.047**	0.05	0.08	0.62	1.05	[0.90, 1.22]	0.533
		post−high school training|*college/university*	0.28	0.08	3.37	1.32	[1.12, 1.55]	**0.001**	0.19	0.10	1.89	1.21	[0.99, 1.48]	0.059	0.29	0.08	3.42	1.34	[1.13, 1.58]	**0.001**
(7)	Stratum	ADM|*ER*	0.13	0.08	1.72	1.14	[0.98, 1.32]	0.086	0.18	0.10	1.78	1.20	[0.98, 1.47]	0.075	0.12	0.08	1.54	1.13	[0.97, 1.32]	0.123
		ICU|*ER*	0.35	0.08	4.60	1.42	[1.22, 1.65]	**<0.001**	0.39	0.14	2.68	1.47	[1.10, 1.96]	**0.007**	0.39	0.08	4.86	1.47	[1.26, 1.72]	**<0.001**
(8)	ISS	total injury severity score	-	-	-	-	-	-	−0.01	0.00	−1.61	0.99	[0.99, 1.00]	0.108	-	-	-	-	-	-
(9)	TBI	uncomplicated mild|*complicated mild*	-	-	-	-	-	-	−0.12	0.09	−1.39	0.88	[0.74, 1.05]	0.164	-	-	-	-	-	-
		moderate|*complicated mild*	-	-	-	-	-	-	−0.18	0.15	−1.21	0.84	[0.63, 1.12]	0.225	-	-	-	-	-	-
		severe|*complicated mild*	-	-	-	-	-	-	−0.38	0.13	−3.00	0.69	[0.54, 0.88]	**0.003**	-	-	-	-	-	-
(11)	Premorbidproblems	none|*emotional*	−0.37	0.12	−3.24	0.69	[0.54, 0.86]	**0.001**	−0.43	0.14	−3.18	0.65	[0.5, 0.84]	**0.001**	−0.36	0.12	−3.04	0.69	[0.55, 0.87]	**0.002**
		physical|*emotional*	−0.25	0.16	−1.59	0.78	[0.57, 1.06]	0.112	−0.38	0.19	−1.98	0.68	[0.47, 0.99]	**0.048**	−0.23	0.17	−1.38	0.80	[0.58, 1.10]	0.168
		concussion|*emotional*	−0.34	0.16	−2.17	0.71	[0.53, 0.97]	**0.030**	−0.39	0.19	−2.06	0.68	[0.47, 0.98]	**0.039**	−0.33	0.16	−2.02	0.72	[0.53, 0.99]	**0.043**
		migraine|*emotional*	−0.13	0.24	−0.54	0.88	[0.56, 1.43]	0.593	−0.07	0.28	−0.25	0.93	[0.55, 1.63]	0.800	−0.14	0.25	−0.56	0.87	[0.55, 1.44]	0.574
		neurological|*emotional*	−0.21	0.18	−1.12	0.81	[0.57, 1.17]	0.262	−0.21	0.22	−0.97	0.81	[0.53, 1.24]	0.331	−0.20	0.19	−1.06	0.82	[0.57, 1.19]	0.288
		mixed|*emotional*	−0.09	0.14	−0.61	0.92	[0.69, 1.21]	0.544	−0.19	0.17	−1.11	0.83	[0.59, 1.15]	0.265	−0.07	0.15	−0.44	0.94	[0.70, 1.25]	0.659
(1:2)	Age: sex	age: male|age:female	0.01	0.00	2.02	1.01	[1.00, 1.01]	**0.043**	0.01	0.00	2.24	1.01	[1.00, 1.02]	**0.025**	0.01	0.00	1.72	1.01	[1.00, 1.01]	0.085

*Note*. Numbers in parenthesis correspond to the numbers in Table 1 and are used for reference in the text; ER = emergency department, ADM = admission, ICU = intensive care unit, TBI = traumatic brain injury, age:sex = interaction between the age and sex groups. The zero model predicts the nonoccurrence of PCS; the count model predicts the average number of PCS. Est. = estimate, model coefficient, S.E. = standard error, *z* = z-value, OR = Odds ratio, CI_95_ = 95% confidence interval [lower, upper]; RR = rate ratio; Bold *p*-values are significant at α = 0.05.

## References

[1] Menon D.K., Schwab K., Wright D.W., Maas A.I. (2010). Position Statement: Definition of Traumatic Brain Injury. Arch. Phys. Med. Rehabil..

[2] Peeters W., van den Brande R., Polinder S., Brazinova A., Steyerberg E.W., Lingsma H.F., Maas A.I.R. (2015). Epidemiology of traumatic brain injury in Europe. Acta Neurochir. (Wien).

[3] Majdan M., Plancikova D., Brazinova A., Rusnak M., Nieboer D., Feigin V., Maas A. (2016). Epidemiology of traumatic brain injuries in Europe: A cross-sectional analysis. Lancet Public Health.

[4] James S.L., Theadom A., Ellenbogen R.G., Bannick M.S., Montjoy-Venning W., Lucchesi L.R., Abbasi N., Abdulkader R., Abraha H.N., Adsuar J.C. (2019). Global, regional, and national burden of traumatic brain injury and spinal cord injury, 1990–2016: A systematic analysis for the Global Burden of Disease Study 2016. Lancet Neurol..

[5] Teasdale G., Jennett B. (1974). Assessment of coma and impaired consciousness. A practical scale. Lancet.

[6] Cassidy J.D., Carroll L., Peloso P., Borg J., von Holst H., Holm L., Kraus J., Coronado V. (2004). Incidence, risk factors and prevention of mild traumatic brain injury: Results of the WHO collaborating centre task force on mild traumatic brain injury. J. Rehabil. Med..

[7] Dewan M.C., Rattani A., Gupta S., Baticulon R.E., Hung Y.-C., Punchak M., Agrawal A., Adeleye A.O., Shrime M.G., Rubiano A.M. (2019). Estimating the global incidence of traumatic brain injury. J. Neurosurg..

[8] Williams D.H., Levin H.S., Eisenberg H.M. (1990). Mild head injury classification. Neurosurgery.

[9] King N.S., Crawford S., Wenden F.J., Moss N.E., Wade D.T. (1995). The Rivermead Post Concussion Symptoms Questionnaire: A measure of symptoms commonly experienced after head injury and its reliability. J. Neurol..

[10] Gordon W.A., Zafonte R., Cicerone K., Cantor J., Brown M., Lombard L., Goldsmith R., Chandna T. (2006). Traumatic Brain Injury Rehabilitation: State of the Science. Am. J. Phys. Med. Rehabil..

[11] Sigurdardottir S., Andelic N., Roe C., Jerstad T., Schanke A.-K. (2009). Post-concussion symptoms after traumatic brain injury at 3 and 12 months post-injury: A prospective study. Brain Inj..

[12] (1994). American Psychiatric Association Diagnostic and Statistical Manual of Mental Disorders.

[13] World Health Organization (2004). ICD-10: International Statistical Classification of Diseases and Related Health Problems, Tenth Revision.

[14] Carroll L., Cassidy J.D., Peloso P., Borg J., von Holst H., Holm L., Paniak C., Pépin M. (2004). Prognosis for mild traumatic brain injury: Results of the who collaborating centre task force on mild traumatic brain injury. J. Rehabil. Med..

[15] Polinder S., Cnossen M.C., Real R.G.L., Covic A., Gorbunova A., Voormolen D.C., Master C.L., Haagsma J.A., Diaz-Arrastia R., von Steinbuechel N. (2018). A Multidimensional Approach to Post-concussion Symptoms in Mild Traumatic Brain Injury. Front. Neurol..

[16] Ryan L.M., Warden D.L. (2003). Post concussion syndrome. Int. Rev. Psychiatry.

[17] Røe C., Sveen U., Alvsåker K., Bautz-Holter E. (2009). Post-concussion symptoms after mild traumatic brain injury: Influence of demographic factors and injury severity in a 1-year cohort study. Disabil. Rehabil..

[18] Kleffelgaard I., Roe C., Soberg H.L., Bergland A. (2012). Associations among self-reported balance problems, post-concussion symptoms and performance-based tests: A longitudinal follow-up study. Disabil. Rehabil..

[19] Arciniegas D.B., Anderson C.A., Topkoff J., McAllister T.W. (2005). Mild traumatic brain injury: A neuropsychiatric approach to diagnosis, evaluation, and treatment. Neuropsychiatr. Dis. Treat..

[20] Evans R.W. (2010). Persistent Post-Traumatic Headache, Postconcussion Syndrome, and Whiplash Injuries: The Evidence for a Non-Traumatic Basis With an Historical Review. Headache J. Head Face Pain.

[21] Rose S.C., Fischer A.N., Heyer G.L. (2015). How long is too long? The lack of consensus regarding the post-concussion syndrome diagnosis. Brain Inj..

[22] Sharp D.J., Jenkins P.O. (2015). Concussion is confusing us all. Pract. Neurol..

[23] Cnossen M.C., van der Naalt J., Spikman J.M., Nieboer D., Yue J.K., Winkler E.A., Manley G.T., von Steinbuechel N., Polinder S., Steyerberg E.W. (2018). Prediction of Persistent Post-Concussion Symptoms after Mild Traumatic Brain Injury. J. Neurotrauma.

[24] Mooney G., Speed J., Sheppard S. (2005). Factors related to recovery after mild traumatic brain injury. Brain Inj..

[25] Voormolen D.C., Cnossen M.C., Polinder S., von Steinbuechel N., Vos P.E., Haagsma J.A. (2018). Divergent Classification Methods of Post-Concussion Syndrome after Mild Traumatic Brain Injury: Prevalence Rates, Risk Factors, and Functional Outcome. J. Neurotrauma.

[26] McLean S.A., Kirsch N.L., Tan-Schriner C.U., Sen A., Frederiksen S., Harris R.E., Maixner W., Maio R.F. (2009). Health status, not head injury, predicts concussion symptoms after minor injury. Am. J. Emerg. Med..

[27] Ponsford J., Nguyen S., Downing M., Bosch M., McKenzie J., Turner S., Chau M., Mortimer D., Gruen R., Knott J. (2019). Factors associated with persistent post-concussion symptoms following mild traumatic brain injury in adults. J. Rehabil. Med..

[28] Levin H.S., Diaz-Arrastia R.R. (2015). Diagnosis, prognosis, and clinical management of mild traumatic brain injury. Lancet Neurol..

[29] Sigurdardottir S., Andelic N., Wehling E., Roe C., Anke A., Skandsen T., Holthe O.O., Jerstad T., Aslaksen P.M., Schanke A.-K. (2015). Neuropsychological Functioning in a National Cohort of Severe Traumatic Brain Injury: Demographic and Acute Injury-Related Predictors. J. Head Trauma Rehabil..

[30] Willer B., Leddy J.J. (2006). Management of concussion and post-concussion syndrome. Curr. Treat. Options Neurol..

[31] Sterr A., Herron K.A., Hayward C., Montaldi D. (2006). Are mild head injuries as mild as we think? Neurobehavioral concomitants of chronic post-concussion syndrome. BMC Neurol..

[32] Meares S., Shores E.A., Taylor A.J., Batchelor J., Bryant R.A., Baguley I.J., Chapman J., Gurka J., Marosszeky J.E. (2011). The prospective course of postconcussion syndrome: The role of mild traumatic brain injury. Neuropsychology.

[33] Meares S., Shores E.A., Taylor A.J., Batchelor J., Bryant R.A., Baguley I.J., Chapman J., Gurka J., Dawson K., Capon L. (2008). Mild traumatic brain injury does not predict acute postconcussion syndrome. J. Neurol. Neurosurg. Psychiatry.

[34] Cohen J. (1983). The Cost of Dichotomization. Appl. Psychol. Meas..

[35] Casson R.J., Farmer L.D. (2014). Understanding and checking the assumptions of linear regression: A primer for medical researchers: Assumptions of linear regression. Clin. Experiment. Ophthalmol..

[36] Iverson G.L. (2019). Network Analysis and Precision Rehabilitation for the Post-concussion Syndrome. Front. Neurol..

[37] Snell D.L., Martin R., Macleod A.D., Surgenor L.J., Siegert R.J., Hay-Smith E.J.C., Melzer T., Hooper G.J., Anderson T. (2018). Untangling chronic pain and post-concussion symptoms: The significance of depression. Brain Inj..

[38] Voormolen D.C., Cnossen M.C., Polinder S., Gravesteijn B.Y., Von Steinbuechel N., Real R.G.L., Haagsma J.A. (2019). Prevalence of post-concussion-like symptoms in the general population in Italy, The Netherlands and the United Kingdom. Brain Inj..

[39] Maas A.I.R., Menon D.K., Steyerberg E.W., Citerio G., Lecky F., Manley G.T., Hill S., Legrand V., Sorgner A. (2015). Collaborative European NeuroTrauma Effectiveness Research in Traumatic Brain Injury (CENTER-TBI): A Prospective Longitudinal Observational Study. Neurosurgery.

[40] Steyerberg E.W., Wiegers E., Sewalt C., Buki A., Citerio G., De Keyser V., Ercole A., Kunzmann K., Lanyon L., Lecky F. (2019). Case-mix, care pathways, and outcomes in patients with traumatic brain injury in CENTER-TBI: A European prospective, multicentre, longitudinal, cohort study. Lancet Neurol..

[41] Acquadro C. (2012). Linguistic Validation Manual for Health Outcome Assessments.

[42] Gennarelli T.A., Wodzin E. (2008). Association for the Advancement of Automotive Medicine Abbreviated Injury Scale 2005: Update 2008.

[43] Eyres S., Carey A., Gilworth G., Neumann V., Tennant A. (2005). Construct validity and reliability of the Rivermead Post-Concussion Symptoms Questionnaire. Clin. Rehabil..

[44] Voormolen D.C., Haagsma J.A., Polinder S., Maas A.I.R., Steyerberg E.W., Vuleković P., Sewalt C.A., Gravesteijn B.Y., Covic A., Andelic N. (2019). Post-Concussion Symptoms in Complicated vs. Uncomplicated Mild Traumatic Brain Injury Patients at Three and Six Months Post-Injury: Results from the CENTER-TBI Study. J. Clin. Med..

[45] Maruta J., Lumba-Brown A., Ghajar J. (2018). Concussion Subtype Identification with the Rivermead Post-concussion Symptoms Questionnaire. Front. Neurol..

[46] Wang Z., Ma S., Wang C.-Y. (2015). Variable selection for zero-inflated and overdispersed data with application to health care demand in Germany: Variable selection for zero-inflated and overdispersed data. Biom. J..

[47] Lambert D. (1992). Zero-Inflated Poisson Regression, with an Application to Defects in Manufacturing. Technometrics.

[48] Hinde J., Demétrio C.G.B. (1998). Overdispersion: Models and estimation. Comput. Stat. Data Anal..

[49] Kleiber C., Zeileis A. (2016). Visualizing Count Data Regressions Using Rootograms. Am. Stat..

[50] Tukey J.W., Bancroft T.A. (1972). Some Graphic and Semigraphic Displays. Statistical Papers in Honor of George W. Snedecor.

[51] Venables W.N., Ripley B.D., Venables W.N. (2002). Modern Applied Statistics with S.

[52] R Core Team (2019). R: A Language and Environment for Statistical Computing.

[53] Zeileis A., Kleiber C. (2018). countreg: Count Data Regression. R Package Version 0.2-1. https://r-forge.r-project.org/projects/countreg/.

[54] Jackman S. (2017). pscl: Classes and Methods for R Developed in the Political Science Computational Laboratory.

[55] Prins M.L., Giza C.C. (2012). Repeat traumatic brain injury in the developing brain. Int. J. Dev. Neurosci..

[56] Field M., Collins M.W., Lovell M.R., Maroon J. (2003). Does age play a role in recovery from sports-related concussion? A comparison of high school and collegiate athletes. J. Pediatr..

[57] Sim A., Terryberry-Spohr L., Wilson K.R. (2008). Prolonged recovery of memory functioning after mild traumatic brain injury in adolescent athletes. J. Neurosurg..

[58] La Fountaine M.F., Hill-Lombardi V., Hohn A.N., Leahy C.L., Testa A.J. (2019). Preliminary Evidence for a Window of Increased Vulnerability to Sustain a Concussion in Females: A Brief Report. Front. Neurol..

[59] Jones H. (2008). Testosterone for the aging male; current evidence and recommended practice. Clin. Interv. Aging.

[60] Ziebell J.M., Rowe R.K., Muccigrosso M.M., Reddaway J.T., Adelson P.D., Godbout J.P., Lifshitz J. (2017). Aging with a traumatic brain injury: Could behavioral morbidities and endocrine symptoms be influenced by microglial priming?. Brain. Behav. Immun..

[61] Barefoot J.C., Mortensen E.L., Helms M.J., Avlund K., Schroll M. (2001). A longitudinal study of gender differences in depressive symptoms from age 50 to 80. Psychol. Aging.

[62] King N. (2014). Permanent post concussion symptoms after mild head injury: A systematic review of age and gender factors. NeuroRehabilitation.

[63] Cutler D.M., Lleras-Muney A. (2010). Understanding differences in health behaviors by education. J. Health Econ..

[64] Wang Y., Chan R., Deng Y. (2006). Examination of postconcussion-like symptoms in healthy university students: Relationships to subjective and objective neuropsychological function performance. Arch. Clin. Neuropsychol..

[65] Stulemeijer M., van der Werf S., Bleijenberg G., Biert J., Brauer J., Vos P.E. (2006). Recovery from mild traumatic brain injury: A focus on fatigue. J. Neurol..

[66] Beaulieu-Bonneau S., Morin C.M. (2012). Sleepiness and fatigue following traumatic brain injury. Sleep Med..

[67] Norrie J., Heitger M., Leathem J., Anderson T., Jones R., Flett R. (2010). Mild traumatic brain injury and fatigue: A prospective longitudinal study. Brain Inj..

[68] Johansson B., Wentzel A.-P., Andréll P., Odenstedt J., Mannheimer C., Rönnbäck L. (2014). Evaluation of dosage, safety and effects of methylphenidate on post-traumatic brain injury symptoms with a focus on mental fatigue and pain. Brain Inj..

[69] Mollayeva T., Kendzerska T., Mollayeva S., Shapiro C.M., Colantonio A., Cassidy J.D. (2014). A systematic review of fatigue in patients with traumatic brain injury: The course, predictors and consequences. Neurosci. Biobehav. Rev..

[70] Potter S., Leigh E., Wade D., Fleminger S. (2006). The Rivermead Post Concussion Symptoms Questionnaire: A confirmatory factor analysis. J. Neurol..

