# Supplementary material for: Influence of Sociodemographic, Premorbid, and Injury-Related Factors on Post-Concussion Symptoms after Traumatic Brain Injury
Source: J Clin Med. 2020 Jun 19;9(6):1931. doi: 10.3390/jcm9061931 (PMC7356324; doi:10.3390/jcm9061931)
Supplement: Supplementary file 1 [file jcm-09-01931-s001.zip › Online_Supplement.pdf]

Table S1. Estimates, odds ratios and rate ratios of the ZINB model. The zero model predicts the probability of *non-occurrence* of PCS; the count model predicts the average number of PCS for the mTBI subsample.

			Zero model						Count model					
No.	Predictors	Levels ∣ reference group	Est.	S.E.	*z*	OR	CI_{95}	*p*	Est.	S.E.	*z*	RR	CI_{95}	*p*
		Intercept	-3.80	0.94	-4.06	0.02	[0.001, 0.14]	**<.001**	1.96	0.25	7.91	7.12	[4.38, 11.58]	**<.001**
(1)	Age	in years	0.02	0.01	1.58	1.02	[1.00, 1.04]	0.115	0.00	0.00	-1.05	1.00	[0.99, 1.00]	0.296
(2)	Sex	male ∣ female	1.03	0.56	1.85	2.81	[0.94, 8.44]	0.065	-0.50	0.21	-2.41	0.61	[0.40, 0.91]	**0.016**
(4)	Employment	part time ∣ full-time	0.04	0.27	0.13	1.04	[0.61, 1.78]	0.894	-0.04	0.10	-0.39	0.96	[0.8, 1.16]	0.697
		in training ∣ full-time	1.13	0.36	3.12	3.08	[1.52, 6.26]	**0.002**	-0.12	0.15	-0.83	0.88	[0.66, 1.19]	0.409
		unemployed ∣ full-time	-0.03	0.40	-0.07	0.97	[0.45, 2.11]	0.943	0.19	0.13	1.54	1.21	[0.95, 1.55]	0.123
		retired ∣ full-time	0.25	0.31	0.80	1.28	[0.70, 2.34]	0.422	-0.18	0.11	-1.60	0.84	[0.68, 1.04]	0.111
(5)	Education	none/primary school ∣ college/university	-0.50	0.31	-1.62	0.61	[0.33, 1.11]	0.106	-0.03	0.11	-0.28	0.97	[0.78, 1.21]	0.778
		secondary/high school ∣ college/university	-0.37	0.23	-1.60	0.69	[0.44, 1.09]	0.109	0.03	0.08	0.39	1.03	[0.88, 1.21]	0.700
		post-high school training ∣ college/university	-0.29	0.25	-1.17	0.75	[0.46, 1.22]	0.243	0.24	0.09	2.56	1.27	[1.06, 1.52]	**0.011**
(7)	Total ISS	total injury severity score	-0.04	0.01	-3.29	0.96	[0.94, 0.98]	**0.001**	0.01	0.00	2.39	1.01	[1.0, 1.01]	**0.017**
(8)	TBI severity	uncomplicated mild ∣ complicated mild	1.00	0.21	4.72	2.73	[1.80, 4.14]	**<.001**	0.04	0.07	0.58	1.04	[0.91, 1.2]	0.560
(10)	Premorbid problems	none ∣ emotional/addiction	1.83	0.67	2.75	6.26	[1.7, 23.11]	**0.006**	-0.15	0.12	-1.27	0.86	[0.68, 1.09]	0.205
		physical ∣ emotional/addiction	0.49	0.82	0.60	1.64	[0.33, 8.19]	0.548	-0.25	0.16	-1.51	0.78	[0.56, 1.08]	0.131
		concussion ∣ emotional/addiction	1.36	0.73	1.85	3.90	[0.93, 16.46]	0.064	-0.22	0.17	-1.30	0.81	[0.58, 1.12]	0.195
		migraine ∣ emotional/addiction	1.79	0.89	2.02	6.02	[1.05, 34.34]	**0.043**	0.14	0.26	0.53	1.15	[0.69, 1.92]	0.598
		neurological ∣ emotional/addiction	1.63	0.77	2.12	5.10	[1.13, 23.04]	**0.034**	0.04	0.20	0.22	1.05	[0.71, 1.55]	0.828
		mixed ∣ emotional/addiction	0.64	0.73	0.88	1.89	[0.45, 7.90]	0.382	0.06	0.14	0.42	1.06	[0.8, 1.4]	0.676
(1:2)	Age : sex	age : male ∣ age : female	-0.01	0.01	-1.09	0.99	[0.97, 1.01]	0.275	0.01	0.00	1.52	1.01	[1, 1.01]	0.128

Note. Numbers in parenthesis correspond to the numbers in Table 1 in the main manuscript and are used for reference in the text; ER = emergency department, ADM = admission, ICU = intensive care unit, TBI = traumatic brain injury, age : sex = interaction between the age and sex groups. The zero model predicts the *non-occurrence* of PCS; the count model predicts the average number of PCS. Est. = estimate, model coefficient, S.E. = standard error, *z* = z-value, OR = Odds ratio, CI_{95} = 95% confidence interval [lower, upper]; RR = rate ratio; Bold *p*-values are significant at $\alpha = .05$.

Table S2. Estimates and rate ratios of negative binomial model for intensity of PCS evaluated with the RPQ total score, RPQ-3, and RPQ-13 score for the mTBI subsample.

			RPQ total score						RPQ-3 score						RPQ-13 score					
No.	Predictors	Levels ∣ reference group	Est.	S.E.	*z*	*RR*	CI_{95}	*p*	Est.	S.E.	*z*	*RR*	CI_{95}	*p*	Est.	S.E.	*z*	*RR*	CI_{95}	*p*
		Intercept	3.25	0.26	12.47	25.90	[15.18, 44.73]	**<.001**	2.12	0.27	7.72	8.31	[4.75, 14.69]	**<.001**	2.94	0.27	10.82	19.00	[10.88, 33.62]	**<.001**
(1)	Age	in years	-0.01	0.00	-1.93	0.99	[0.98, 1.00]	0.053	-0.01	0.00	-3.38	0.99	[0.98, 0.99]	**0.001**	-0.01	0.00	-1.41	0.99	[0.99, 1.00]	0.159
(2)	Sex	male ∣ female	-0.61	0.22	-2.80	0.54	[0.35, 0.83]	**0.005**	-0.90	0.24	-3.79	0.41	[0.26, 0.65]	**<.001**	-0.53	0.23	-2.33	0.59	[0.37, 0.92]	**0.020**
(4)	Employment	part time ∣ full-time	-0.04	0.11	-0.34	0.96	[0.78, 1.20]	0.736	-0.17	0.12	-1.38	0.85	[0.67, 1.08]	0.168	-0.01	0.11	-0.10	0.99	[0.80, 1.24]	0.924
		in training ∣ full-time	-0.41	0.15	-2.76	0.66	[0.50, 0.89]	**0.006**	-0.42	0.17	-2.51	0.66	[0.48, 0.92]	**0.012**	-0.41	0.15	-2.67	0.66	[0.49, 0.90]	**0.008**
		unemployed ∣ full-time	0.17	0.14	1.15	1.18	[0.89, 1.58]	0.250	0.14	0.16	0.88	1.15	[0.85, 1.57]	0.377	0.17	0.15	1.16	1.19	[0.89, 1.61]	0.247
		retired ∣ full-time	-0.18	0.12	-1.47	0.84	[0.66, 1.06]	0.141	-0.11	0.14	-0.80	0.90	[0.69, 1.17]	0.422	-0.19	0.12	-1.49	0.83	[0.65, 1.06]	0.138
(5)	Education	none/primary school ∣ college/university	0.11	0.12	0.91	1.11	[0.88, 1.40]	0.362	0.28	0.13	2.16	1.33	[1.02, 1.73]	**0.031**	0.07	0.12	0.56	1.07	[0.84, 1.36]	0.577
		at least secondary/high school ∣ college/university	0.14	0.09	1.59	1.15	[0.97, 1.37]	0.112	0.27	0.10	2.65	1.30	[1.07, 1.59]	**0.008**	0.12	0.09	1.29	1.13	[0.94, 1.35]	0.197
		post-high school training ∣ college/university	0.26	0.10	2.61	1.30	[1.06, 1.58]	**0.009**	0.14	0.11	1.20	1.15	[0.92, 1.44]	0.229	0.28	0.10	2.72	1.33	[1.08, 1.63]	**0.007**
(7)	ISS	total injury severity score	0.01	0.00	2.97	1.01	[1.00, 1.02]	**0.003**	-	-	-	-	-	-	0.01	0.00	3.12	1.01	[1.00, 1.02]	**0.002**
(8)	TBI severity	uncomplicated mild ∣ complicated mild	-0.14	0.08	-1.85	0.87	[0.75, 1.01]	0.065	-0.22	0.08	-2.71	0.81	[0.69, 0.94]	**0.007**	-0.13	0.08	-1.65	0.88	[0.75, 1.03]	0.100
(9)	LOC	no ∣ yes	0.15	0.12	1.25	1.16	[0.92, 1.47]	0.211	-	-	-	-	-	-	0.14	0.12	1.14	1.15	[0.90, 1.48]	0.254
(10)	Premorbid problems	none ∣ emotional/addiction	-0.43	0.15	-2.91	0.65	[0.48, 0.86]	**0.004**	-0.48	0.16	-3.02	0.62	[0.45, 0.84]	**0.003**	-0.42	0.15	-2.72	0.66	[0.48, 0.88]	**0.007**
		physical ∣ emotional/addiction	-0.24	0.20	-1.23	0.79	[0.53, 1.15]	0.219	-0.39	0.22	-1.80	0.68	[0.44, 1.03]	0.072	-0.21	0.20	-1.05	0.81	[0.54, 1.20]	0.296
		concussion ∣ emotional/addiction	-0.37	0.19	-1.93	0.69	[0.47, 1.00]	0.053	-0.42	0.21	-1.99	0.66	[0.43, 0.99]	**0.047**	-0.36	0.20	-1.79	0.70	[0.47, 1.03]	0.074
		migraine ∣ emotional/addiction	-0.11	0.30	-0.37	0.89	[0.51, 1.66]	0.708	-0.01	0.32	-0.05	0.99	[0.54, 1.88]	0.963	-0.14	0.31	-0.44	0.87	[0.48, 1.66]	0.658
		neurological ∣ emotional/addiction	-0.23	0.23	-0.98	0.80	[0.51, 1.26]	0.326	-0.15	0.25	-0.61	0.86	[0.53, 1.41]	0.542	-0.23	0.24	-0.98	0.79	[0.50, 1.27]	0.329
		mixed ∣ emotional/addiction	-0.01	0.18	-0.07	0.99	[0.69, 1.39]	0.947	-0.18	0.19	-0.97	0.83	[0.57, 1.21]	0.334	0.02	0.18	0.09	1.02	[0.70, 1.45]	0.927
(1:2)	Age : sex	age : male ∣ age : female	0.01	0.00	2.07	1.01	[1.00, 1.02]	**0.038**	0.01	0.00	2.15	1.01	[1.00, 1.02]	**0.031**	0.01	0.00	1.76	1.01	[1.00, 1.02]	0.078
(2:10)	Sex : LOC	male : LOC ∣ female : LOC	-0.23	0.15	-1.52	0.80	[0.59, 1.07]	0.129	-	-	-	-	-	-	-0.24	0.16	-1.55	0.79	[0.58, 1.07]	0.121

Note. Numbers in parenthesis correspond to the numbers in Table 1 and are used for reference in the text; ER = emergency department, ADM = admission, ICU = intensive care unit, TBI = traumatic brain injury, LOC = loss of consciousness, age : sex = interaction between the age and sex groups. The zero model predicts the *non-occurrence* of PCS; the count model predicts the average number of PCS. Est. = estimate, model coefficient, S.E. = standard error, z = z-value, OR = Odds ratio, CI_{95} = 95% confidence interval [lower, upper]; RR = rate ratio; **bold** p-values are significant at α = .05.
