# Supplementary material for: Influence of Sociodemographic, Premorbid, and Injury-Related Factors on Post-Concussion Symptoms after Traumatic Brain Injury
Source: J Clin Med. 2020 Jun 19;9(6):1931. doi: 10.3390/jcm9061931 (PMC7356324; doi:10.3390/jcm9061931)
Supplement: Supplementary file 1 [file jcm-09-01931-s001.zip › Online_Supplement_List_of_Investigators.pdf]

The CENTER-TBI participants and investigators:

Cecilia Åkerlund[1], Krisztina Amrein[2], Nada Andelic[3], Lasse Andreassen[4], Audny Anke[5], Anna Antoni[6], Gérard Audibert[7], Philippe Azouvi[8], Maria Luisa Azzolini[9], Ronald Bartels[10], Pál Barzó[11], Romuald Beauvais[12], Ronny Beer[13], Bo-Michael Bellander[14], Antonio Belli[15], Habib Benali[16], Maurizio Berardino[17], Luigi Beretta[9], Morten Blaabjerg[18], Peter Bragge[19], Alexandra Brazinova[20], Vibeke Brinck[21], Joanne Brooker[22], Camilla Brorsson[23], Andras Buki[24], Monika Bullinger[25], Manuel Cabeleira[26], Alessio Caccioppola[27], Emiliana Calappi [27], Maria Rosa Calvi[9], Peter Cameron[28], Guillermo Carbayo Lozano[29], Marco Carbonara[27], Simona Cavallo[17], Giorgio Chevallard[30], Arturo Chieregato[30], Giuseppe Citerio[31, 32], Iris Ceyisakar[33], Hans Clusmann[34], Mark Coburn[35], Jonathan Coles[36], Jamie D. Cooper[37], Marta Correia[38], Amra Čović [39], Nicola Curry[40], Endre Czeiter[24], Marek Czosnyka[26], Claire Dahyot-Fizelier[41], Paul Dark[42], Helen Dawes[43], Véronique De Keyser[44], Vincent Degos[16], Francesco Della Corte[45], Hugo den Boogert[10], Bart Depreitere[46], Đula Đilvesi [47], Abhishek Dixit[48], Emma Donoghue[22], Jens Dreier[49], Guy-Loup Dulière[50], Ari Ercole[48], Patrick Esser[43], Erzsébet Ezer[51], Martin Fabricius[52], Valery L. Feigin[53], Kelly Foks[54], Shirin Frisvold[55], Alex Furmanov[56], Pablo Gagliardo[57], Damien Galanaud[16], Dashiell Gantner[28], Guoyi Gao[58], Pradeep George[59], Alexandre Ghuysen[60], Lelde Giga[61], Ben Glocker[62], Jagoš Golubovic[47], Pedro A. Gomez [63], Johannes Gratz[64], Benjamin Gravesteijn[33], Francesca Grossi[45], Russell L. Gruen[65], Deepak Gupta[66], Juanita A. Haagsma[33], Iain Haitsma[67], Raimund Helbok[13], Eirik Helseth[68], Lindsay Horton [69], Jilske Huijben[33], Peter J. Hutchinson[70], Bram Jacobs[71], Stefan Jankowski[72], Mike Jarrett[21], Ji-yao Jiang[58], Faye Johnson[73], Kelly Jones[53], Mladen Karan[47], Angelos G. Kolias[70], Erwin Kompanje[74], Daniel Kondziella[52], Evgenios Koraropoulos[48], Lars-Owe Koskinen[75], Noémi Kovács[76], Ana Kowark[35], Alfonso Lagares[63], Linda Lanyon[59], Steven Laureys[77], Fiona Lecky[78, 79], Didier Ledoux[77], Rolf Lefering[80], Valerie Legrand[81], Aurelie Lejeune[82], Leon Levi[83], Roger Lightfoot[84], Hester Lingsma[33], Andrew I.R. Maas[44], Ana M. Castaño-León[63], Marc Maegele[85], Marek Majdan[20], Alex Manara[86], Geoffrey Manley[87], Costanza Martino[88], Hugues Maréchal[50], Julia Mattern[89], Catherine McMahon[90], Béla Melegh[91], David Menon[48], Tomas Menovsky[44], Ana Mikolic[33], Benoit Misset[77], Visakh Muraleedharan[59], Lynnette Murray[28], Ancuta Negru[92], David Nelson[1], Virginia Newcombe[48], Daan Nieboer[33], József Nyirádi[2], Otesile Olubukola[78], Matej Oresic[93], Fabrizio Ortolano[27], Aarno Palotie[94, 95, 96], Paul M. Parizel[97], Jean-François Payen[98], Natascha Perera[12], Vincent Perlbarg[16], Paolo Persona[99], Wilco Peul[100], Anna Piippo-Karjalainen[101], Matti Pirinen[94], Horia Ples[92], Suzanne Polinder[33], Inigo Pomposo[29], Jussi P. Posti [102], Louis Puybasset[103], Andreea Radoi [104], Arminas Ragauskas[105], Rahul Raj[101], Malinka Rambadagalla[106], Jonathan Rhodes[107], Sylvia Richardson[108], Sophie Richter[48], Samuli Ripatti[94], Saulius Rocka[105], Cecilie Roe[109], Olav Roise[110,111], Jonathan Rosand[112], Jeffrey V. Rosenfeld[113], Christina Rosenlund[114], Guy Rosenthal[56], Rolf Rossaint[35], Sandra Rossi[99], Daniel Rueckert[62], Martin Rusnák[115], Juan Sahuquillo[104], Oliver Sakowitz[89, 116], Renan Sanchez-Porras[116], Janos Sandor[117], Nadine Schäfer[80], Silke Schmidt[118], Herbert Schoechl[119], Guus Schoonman[120], Rico Frederik Schou[121], Elisabeth Schwendenwein[6], Charlie Sewalt[33], Toril Skandsen[122, 123] , Peter Smielewski[26], Abayomi Sorinola[124], Emmanuel Stamatakis[48], Simon Stanworth[40], Robert Stevens[125], William Stewart[126], Ewout W. Steyerberg[33, 127], Nino Stocchetti[128], Nina Sundström[129], Anneliese Synnot[22, 130], Riikka Takala[131], Viktória Tamás[124], Tomas Tamosuitis[132], Mark Steven Taylor[20], Braden Te Ao[53], Olli Tenovuo[102], Alice Theadom[53], Matt Thomas[86], Dick Tibboel[133], Marjolein Timmers[74], Christos Tolias[134], Tony Trapani[28], Cristina Maria Tudora[92], Peter Vajkoczy [135], Shirley Vallance[28], Egils Valeinis[61], Zoltán Vámos[51], Mathieu van der Jagt[136], Gregory Van der Steen[44], Joukje van der Naalt[71], Jeroen T.J.M. van Dijck [100], Thomas A. van Essen[100], Wim Van Hecke[137], Caroline van Heugten[138], Dominique Van Praag[139], Thijs Vande Vyvere[137], Roel P. J. van Wijk[100], Alessia Vargiolu[32], Emmanuel Vega[82], Kimberley Velt[33], Jan Verheyden[137], Paul M. Vespa[140], Anne Vik[122, 141], Rimantas Vilcinis[132], Victor Volovici[67], Nicole von Steinbüchel[39], Daphne Voormolen[33], Petar Vulekovic[47], Kevin K.W. Wang[142], Eveline Wiegers[33], Guy Williams[48], Lindsay Wilson[69], Stefan Winzeck[48], Stefan Wolf[143], Zhihui Yang[142], Peter Ylén[144], Alexander Younsi[89], Frederick A. Zeiler[48,145], Veronika Zelinkova[20], Agate Ziverte[61] , Tommaso Zoerle[27]

[1] Department of Physiology and Pharmacology, Section of Perioperative Medicine and Intensive Care, Karolinska Institutet, Stockholm, Sweden

[2] János Szentágothai Research Centre, University of Pécs, Pécs, Hungary

[3] Division of Surgery and Clinical Neuroscience, Department of Physical Medicine and Rehabilitation, Oslo University Hospital and University of Oslo, Oslo, Norway

[4] Department of Neurosurgery, University Hospital Northern Norway, Tromso, Norway

[5] Department of Physical Medicine and Rehabilitation, University Hospital Northern Norway, Tromso, Norway

[6] Trauma Surgery, Medical University Vienna, Vienna, Austria

[7] Department of Anesthesiology & Intensive Care, University Hospital Nancy, Nancy, France

[8] Raymond Poincare hospital, Assistance Publique – Hopitaux de Paris, Paris, France

[9] Department of Anesthesiology & Intensive Care, S Raffaele University Hospital, Milan, Italy

[10] Department of Neurosurgery, Radboud University Medical Center, Nijmegen, The Netherlands

[11] Department of Neurosurgery, University of Szeged, Szeged, Hungary

[12] International Projects Management, ARTTIC, Munchen, Germany

[13] Department of Neurology, Neurological Intensive Care Unit, Medical University of Innsbruck, Innsbruck, Austria

[14] Department of Neurosurgery & Anesthesia & intensive care medicine, Karolinska University Hospital, Stockholm, Sweden

[15] NIHR Surgical Reconstruction and Microbiology Research Centre, Birmingham, UK

[16] Anesthesie-Réanimation, Assistance Publique – Hopitaux de Paris, Paris, France

[17] Department of Anesthesia & ICU, AOU Città della Salute e della Scienza di Torino - Orthopedic and Trauma Center, Torino, Italy

[18] Department of Neurology, Odense University Hospital, Odense, Denmark

[19] BehaviourWorks Australia, Monash Sustainability Institute, Monash University, Victoria, Australia

[20] Department of Public Health, Faculty of Health Sciences and Social Work, Trnava University, Trnava, Slovakia

[21] Quesgen Systems Inc., Burlingame, California, USA

[22] Australian & New Zealand Intensive Care Research Centre, Department of Epidemiology and Preventive Medicine, School of Public Health and Preventive Medicine, Monash University, Melbourne, Australia

[23] Department of Surgery and Perioperative Science, Umeå University, Umeå, Sweden
[24] Department of Neurosurgery, Medical School, University of Pécs, Hungary and Neurotrauma Research Group, János Szentágothai Research Centre, University of Pécs, Hungary
[25] Department of Medical Psychology, Universitätsklinikum Hamburg-Eppendorf, Hamburg, Germany
[26] Brain Physics Lab, Division of Neurosurgery, Dept of Clinical Neurosciences, University of Cambridge, Addenbrooke's Hospital, Cambridge, UK
[27] Neuro ICU, Fondazione IRCCS Cà Granda Ospedale Maggiore Policlinico, Milan, Italy
[28] ANZIC Research Centre, Monash University, Department of Epidemiology and Preventive Medicine, Melbourne, Victoria, Australia
[29] Department of Neurosurgery, Hospital of Cruces, Bilbao, Spain
[30] NeuroIntensive Care, Niguarda Hospital, Milan, Italy
[31] School of Medicine and Surgery, Università Milano Bicocca, Milano, Italy
[32] NeuroIntensive Care, ASST di Monza, Monza, Italy
[33] Department of Public Health, Erasmus Medical Center-University Medical Center, Rotterdam, The Netherlands
[34]Department of Neurosurgery, Medical Faculty RWTH Aachen University, Aachen, Germany
[35] Department of Anaesthesiology, University Hospital of Aachen, Aachen, Germany
[36] Department of Anesthesia & Neurointensive Care, Cambridge University Hospital NHS Foundation Trust, Cambridge, UK
[37] School of Public Health & PM, Monash University and The Alfred Hospital, Melbourne, Victoria, Australia
[38] Radiology/MRI department, MRC Cognition and Brain Sciences Unit, Cambridge, UK
[39] Institute of Medical Psychology and Medical Sociology, Universitätsmedizin Göttingen, Göttingen, Germany
[40] Oxford University Hospitals NHS Trust, Oxford, UK
[41] Intensive Care Unit, CHU Poitiers, Potiers, France
[42] University of Manchester NIHR Biomedical Research Centre, Critical Care Directorate, Salford Royal Hospital NHS Foundation Trust, Salford, UK
[43] Movement Science Group, Faculty of Health and Life Sciences, Oxford Brookes University, Oxford, UK
[44] Department of Neurosurgery, Antwerp University Hospital and University of Antwerp, Edegem, Belgium
[45] Department of Anesthesia & Intensive Care, Maggiore Della Carità Hospital, Novara, Italy
[46] Department of Neurosurgery, University Hospitals Leuven, Leuven, Belgium
[47] Department of Neurosurgery, Clinical centre of Vojvodina, Faculty of Medicine, University of Novi Sad, Novi Sad, Serbia
[48] Division of Anaesthesia, University of Cambridge, Addenbrooke's Hospital, Cambridge, UK
[49] Center for Stroke Research Berlin, Charité – Universitätsmedizin Berlin, corporate member of Freie Universität Berlin, Humboldt-Universität zu Berlin, and Berlin Institute of Health, Berlin, Germany
[50] Intensive Care Unit, CHR Citadelle, Liège, Belgium
[51] Department of Anaesthesiology and Intensive Therapy, University of Pécs, Pécs, Hungary
[52] Departments of Neurology, Clinical Neurophysiology and Neuroanesthesiology, Region Hovedstaden Rigshospitalet, Copenhagen, Denmark
[53] National Institute for Stroke and Applied Neurosciences, Faculty of Health and Environmental Studies, Auckland University of Technology, Auckland, New Zealand
[54] Department of Neurology, Erasmus MC, Rotterdam, the Netherlands
[55] Department of Anesthesiology and Intensive care, University Hospital Northern Norway, Tromso, Norway
[56] Department of Neurosurgery, Hadassah-hebrew University Medical center, Jerusalem, Israel
[57] Fundación Instituto Valenciano de Neurorrehabilitación (FIVAN), Valencia, Spain
[58] Department of Neurosurgery, Shanghai Renji hospital, Shanghai Jiaotong University/school of medicine, Shanghai, China
[59] Karolinska Institutet, INCF International Neuroinformatics Coordinating Facility, Stockholm, Sweden
[60] Emergency Department, CHU, Liège, Belgium
[61] Neurosurgery clinic, Pauls Stradins Clinical University Hospital, Riga, Latvia
[62] Department of Computing, Imperial College London, London, UK
[63] Department of Neurosurgery, Hospital Universitario 12 de Octubre, Madrid, Spain
[64] Department of Anesthesia, Critical Care and Pain Medicine, Medical University of Vienna, Austria
[65] College of Health and Medicine, Australian National University, Canberra, Australia
[66] Department of Neurosurgery, Neurosciences Centre & JPN Apex trauma centre, All India Institute of Medical Sciences, New Delhi-110029, India
[67] Department of Neurosurgery, Erasmus MC, Rotterdam, the Netherlands
[68] Department of Neurosurgery, Oslo University Hospital, Oslo, Norway
[69] Division of Psychology, University of Stirling, Stirling, UK
[70] Division of Neurosurgery, Department of Clinical Neurosciences, Addenbrooke's Hospital & University of Cambridge, Cambridge, UK
[71] Department of Neurology, University of Groningen, University Medical Center Groningen, Groningen, Netherlands
[72] Neurointensive Care, Sheffield Teaching Hospitals NHS Foundation Trust, Sheffield, UK
[73] Salford Royal Hospital NHS Foundation Trust Acute Research Delivery Team, Salford, UK
[74] Department of Intensive Care and Department of Ethics and Philosophy of Medicine, Erasmus Medical Center, Rotterdam, The Netherlands
[75] Department of Clinical Neuroscience, Neurosurgery, Umeå University, Umeå, Sweden
[76] Hungarian Brain Research Program - Grant No. KTIA_13_NAP-A-II/8, University of Pécs, Pécs, Hungary
[77] Cyclotron Research Center, University of Liège, Liège, Belgium

[78] Centre for Urgent and Emergency Care Research (CURE), Health Services Research Section, School of Health and Related Research (ScHARR), University of Sheffield, Sheffield, UK
[79] Emergency Department, Salford Royal Hospital, Salford UK
[80] Institute of Research in Operative Medicine (IFOM), Witten/Herdecke University, Cologne, Germany
[81] VP Global Project Management CNS, ICON, Paris, France
[82] Department of Anesthesiology-Intensive Care, Lille University Hospital, Lille, France
[83] Department of Neurosurgery, Rambam Medical Center, Haifa, Israel
[84] Department of Anesthesiology & Intensive Care, University Hospitals Southhampton NHS Trust, Southhampton, UK
[85] Cologne-Merheim Medical Center (CMMC), Department of Traumatology, Orthopedic Surgery and Sportmedicine, Witten/Herdecke University, Cologne, Germany
[86] Intensive Care Unit, Southmead Hospital, Bristol, Bristol, UK
[87] Department of Neurological Surgery, University of California, San Francisco, California, USA
[88] Department of Anesthesia & Intensive Care,M. Bufalini Hospital, Cesena, Italy
[89] Department of Neurosurgery, University Hospital Heidelberg, Heidelberg, Germany
[90] Department of Neurosurgery, The Walton centre NHS Foundation Trust, Liverpool, UK
[91] Department of Medical Genetics, University of Pécs, Pécs, Hungary
[92] Department of Neurosurgery, Emergency County Hospital Timisoara, Timisoara, Romania
[93] School of Medical Sciences, Örebro University, Örebro, Sweden
[94] Institute for Molecular Medicine Finland, University of Helsinki, Helsinki, Finland
[95] Analytic and Translational Genetics Unit, Department of Medicine; Psychiatric & Neurodevelopmental Genetics Unit, Department of Psychiatry; Department of Neurology, Massachusetts General Hospital, Boston, MA, USA
[96] Program in Medical and Population Genetics; The Stanley Center for Psychiatric Research, The Broad Institute of MIT and Harvard, Cambridge, MA, USA
[97] Department of Radiology, University of Antwerp, Edegem, Belgium
[98] Department of Anesthesiology & Intensive Care, University Hospital of Grenoble, Grenoble, France
[99] Department of Anesthesia & Intensive Care, Azienda Ospedaliera Università di Padova, Padova, Italy
[100] Dept. of Neurosurgery, Leiden University Medical Center, Leiden, The Netherlands and Dept. of Neurosurgery, Medical Center Haaglanden, The Hague, The Netherlands
[101] Department of Neurosurgery, Helsinki University Central Hospital
[102] Division of Clinical Neurosciences, Department of Neurosurgery and Turku Brain Injury Centre, Turku University Hospital and University of Turku, Turku, Finland
[103] Department of Anesthesiology and Critical Care, Pitié -Salpêtrière Teaching Hospital, Assistance Publique, Hôpitaux de Paris and University Pierre et Marie Curie, Paris, France
[104] Neurotraumatology and Neurosurgery Research Unit (UNINN), Vall d'Hebron Research Institute, Barcelona, Spain
[105] Department of Neurosurgery, Kaunas University of technology and Vilnius University, Vilnius, Lithuania
[106] Department of Neurosurgery, Rezekne Hospital, Latvia
[107] Department of Anesthesia, Critical Care & Pain Medicine NHS Lothian & University of Edinburg, Edinburgh, UK
[108] Director, MRC Biostatistics Unit, Cambridge Institute of Public Health, Cambridge, UK
[109] Department of Physical Medicine and Rehabilitation, Oslo University Hospital/University of Oslo, Oslo, Norway
[110] Division of Orthopedics, Oslo University Hospital, Oslo, Norway
[111] Institute of Clinical Medicine, Faculty of Medicine, University of Oslo, Oslo, Norway
[112] Broad Institute, Cambridge MA Harvard Medical School, Boston MA, Massachusetts General Hospital, Boston MA, USA
[113] National Trauma Research Institute, The Alfred Hospital, Monash University, Melbourne, Victoria, Australia
[114] Department of Neurosurgery, Odense University Hospital, Odense, Denmark
[115] International Neurotrauma Research Organization, Vienna, Austria
[116] Klinik für Neurochirurgie, Klinikum Ludwigsburg, Ludwigsburg, Germany
[117] Division of Biostatistics and Epidemiology, Department of Preventive Medicine, University of Debrecen, Debrecen, Hungary
[118] Department Health and Prevention, University Greifswald, Greifswald, Germany
[119] Department of Anesthesiology and Intensive Care, AUVA Trauma Hospital, Salzburg, Austria
[120] Department of Neurology, Elisabeth-TweeSteden Ziekenhuis, Tilburg, the Netherlands
[121] Department of Neuroanesthesia and Neurointensive Care, Odense University Hospital, Odense, Denmark
[122] Department of Neuromedicine and Movement Science, Norwegian University of Science and Technology, NTNU, Trondheim, Norway
[123] Department of Physical Medicine and Rehabilitation, St. Olavs Hospital, Trondheim University Hospital, Trondheim, Norway
[124] Department of Neurosurgery, University of Pécs, Pécs, Hungary
[125] Division of Neuroscience Critical Care, John Hopkins University School of Medicine, Baltimore, USA
[126] Department of Neuropathology, Queen Elizabeth University Hospital and University of Glasgow, Glasgow, UK
[127] Dept. of Department of Biomedical Data Sciences, Leiden University Medical Center, Leiden, The Netherlands
[128] Department of Pathophysiology and Transplantation, Milan University, and Neuroscience ICU, Fondazione IRCCS Cà Granda Ospedale Maggiore Policlinico, Milano, Italy
[129] Department of Radiation Sciences, Biomedical Engineering, Umeå University, Umeå, Sweden
[130] Cochrane Consumers and Communication Review Group, Centre for Health Communication and Participation, School of Psychology and Public Health, La Trobe University, Melbourne, Australia
[131] Perioperative Services, Intensive Care Medicine and Pain Management, Turku University Hospital and University of Turku, Turku, Finland

[132] Department of Neurosurgery, Kaunas University of Health Sciences, Kaunas, Lithuania
[133] Intensive Care and Department of Pediatric Surgery, Erasmus Medical Center, Sophia Children's Hospital, Rotterdam, The Netherlands
[134] Department of Neurosurgery, Kings college London, London, UK
[135] Neurologie, Neurochirurgie und Psychiatrie, Charité – Universitätsmedizin Berlin, Berlin, Germany
[136] Department of Intensive Care Adults, Erasmus MC– University Medical Center Rotterdam, Rotterdam, the Netherlands
[137] icoMetrix NV, Leuven, Belgium
[138] Movement Science Group, Faculty of Health and Life Sciences, Oxford Brookes University, Oxford, UK
[139] Psychology Department, Antwerp University Hospital, Edegem, Belgium
[140] Director of Neurocritical Care, University of California, Los Angeles, USA
[141] Department of Neurosurgery, St.Olavs Hospital, Trondheim University Hospital, Trondheim, Norway
[142] Department of Emergency Medicine, University of Florida, Gainesville, Florida, USA
[143] Department of Neurosurgery, Charité – Universitätsmedizin Berlin, corporate member of Freie Universität Berlin, Humboldt-Universität zu Berlin, and Berlin Institute of Health, Berlin, Germany
[144] VTT Technical Research Centre, Tampere, Finland
[145] Section of Neurosurgery, Department of Surgery, Rady Faculty of Health Sciences, University of Manitoba, Winnipeg, MB, Canada
